# NbPsbO1 Interacts Specifically with the *Bamboo Mosaic Virus* (BaMV) Subgenomic RNA (sgRNA) Promoter and Is Required for Efficient BaMV sgRNA Transcription

**DOI:** 10.1128/JVI.00831-21

**Published:** 2021-09-27

**Authors:** Ying Wen Huang, Chu I Sun, Chung Chi Hu, Ching Hsiu Tsai, Menghsiao Meng, Na Sheng Lin, Yau Heiu Hsu

**Affiliations:** a Graduate Institute of Biotechnology, National Chung Hisng University, Taichung, Taiwan; b Advanced Plant Biotechnology Center, National Chung Hsing University, Taichung, Taiwan; c Institute of Plant and Microbial Biology, Academia Sinica, Taipei, Taiwan; University of Maryland, College Park

**Keywords:** *Bamboo mosaic virus*, BaMV, chloroplast, *Nicotiana benthamiana* PsbO1, replication complexes, subgenomic RNA transcription

## Abstract

Many positive-strand (+) RNA viruses produce subgenomic RNAs (sgRNAs) in the infection cycle through the combined activities of viral replicase and host proteins. However, knowledge about host proteins involved in direct sgRNA promoter recognition is limited. Here, in the partially purified replicase complexes from *Bamboo mosaic virus* (BaMV)-infected tissue, we have identified the Nicotiana benthamiana photosystem II oxygen-evolving complex protein, NbPsbO1, which specifically interacted with the promoter of sgRNA but not that of genomic RNA (gRNA). Silencing of *NbPsbO1* expression suppressed BaMV accumulation in N. benthamiana protoplasts without affecting viral gRNA replication. Overexpression of wild-type NbPsbO1 stimulated BaMV sgRNA accumulation. Fluorescent microscopy examination revealed that the fluorescence associated with NbPsbO1 was redistributed from chloroplast granal thylakoids to stroma in BaMV-infected cells. Overexpression of a mislocalized mutant of NbPsbO1, dTPPsbO1-T7, inhibited BaMV RNA accumulation in N. benthamiana, whereas overexpression of an NbPsbO1 derivative, sPsbO1-T7, designed to be targeted to chloroplast stroma, upregulated the sgRNA level. Furthermore, depletion of NbPsbO1 in BaMV RdRp preparation significantly inhibited sgRNA synthesis *in vitro* but exerted no effect on (+) or (−) gRNA synthesis, which indicates that NbPsbO1 is required for efficient sgRNA synthesis. These results reveal a novel role for NbPsbO1 in the selective enhancement of BaMV sgRNA transcription, most likely via direct interaction with the sgRNA promoter.

**IMPORTANCE** Production of subgenomic RNAs (sgRNAs) for efficient translation of downstream viral proteins is one of the major strategies adapted for viruses that contain a multicistronic RNA genome. Both viral genomic RNA (gRNA) replication and sgRNA transcription rely on the combined activities of viral replicase and host proteins, which recognize promoter regions for the initiation of RNA synthesis. However, compared to the *cis*-acting elements involved in the regulation of sgRNA synthesis, the host factors involved in sgRNA promoter recognition mostly remain to be elucidated. Here, we found a chloroplast protein, NbPsbO1, which specifically interacts with *Bamboo mosaic virus* (BaMV) sgRNA promoter. We showed that NbPsbO1 is relocated to the BaMV replication site in BaMV-infected cells and demonstrated that NbPsbO1 is required for efficient BaMV sgRNA transcription but exerts no effect on gRNA replication. This study provides a new insight into the regulating mechanism of viral gRNA and sgRNA synthesis.

## INTRODUCTION

Positive-strand (+)RNA viruses cause widespread diseases in humans, animals, and plants. Although infecting hosts of different kingdoms, (+)RNA viruses undergo a similar replication cycle that consists of several distinct steps, including (i) recruitment of a (+)RNA template and switching from translation to replication mode, (ii) transportation of viral RNA-dependent RNA polymerase (RdRp) to the replication site, (iii) rearrangement of intracellular membranes and assembly of the viral replication complexes (VRCs), and (iv) synthesis of negative-strand (−) viral RNAs followed by that of (+) viral RNAs, or (v) for some (+)RNA viruses, also synthesis of one or more subgenomic RNAs (sgRNAs) via a (−) viral RNA intermediate (1). Because of the limited coding capacity, viruses require a multitude of host factors to support or regulate their biological functions in each of these steps (2–4).

Many (+)RNA viruses possess multicistronic genomes that produce sgRNAs to serve as messengers, allowing for the translation of downstream open reading frames (ORFs) in monocistronic host environments (5). The (−) viral RNA intermediates harbor *cis*-acting elements containing functional sequences and structures recognized by replication complexes for initiation of transcription (6–8). The efficiency and timing of sgRNA synthesis allow for quantitative and temporal control of viral protein expression. Therefore, knowledge of the regulatory mechanism of gRNA and sgRNA synthesis, including identifying the *cis*-acting elements for RdRp recognition and the host proteins involved in the replication complexes, is of vital importance for the management of viral diseases and the development of virus-based applications in biotechnology.

*In vitro* RdRp systems are commonly used to investigate the components of host and viral proteins associated with RdRp complexes and for defining the minimal requirement of *cis*-acting RNA elements for replication (9–11). Efforts toward unraveling the interactions between viruses and their host cells make a great contribution to our understanding of viral infections, thus providing a valuable resource for the development of antiviral strategies.

**FIG 1:**
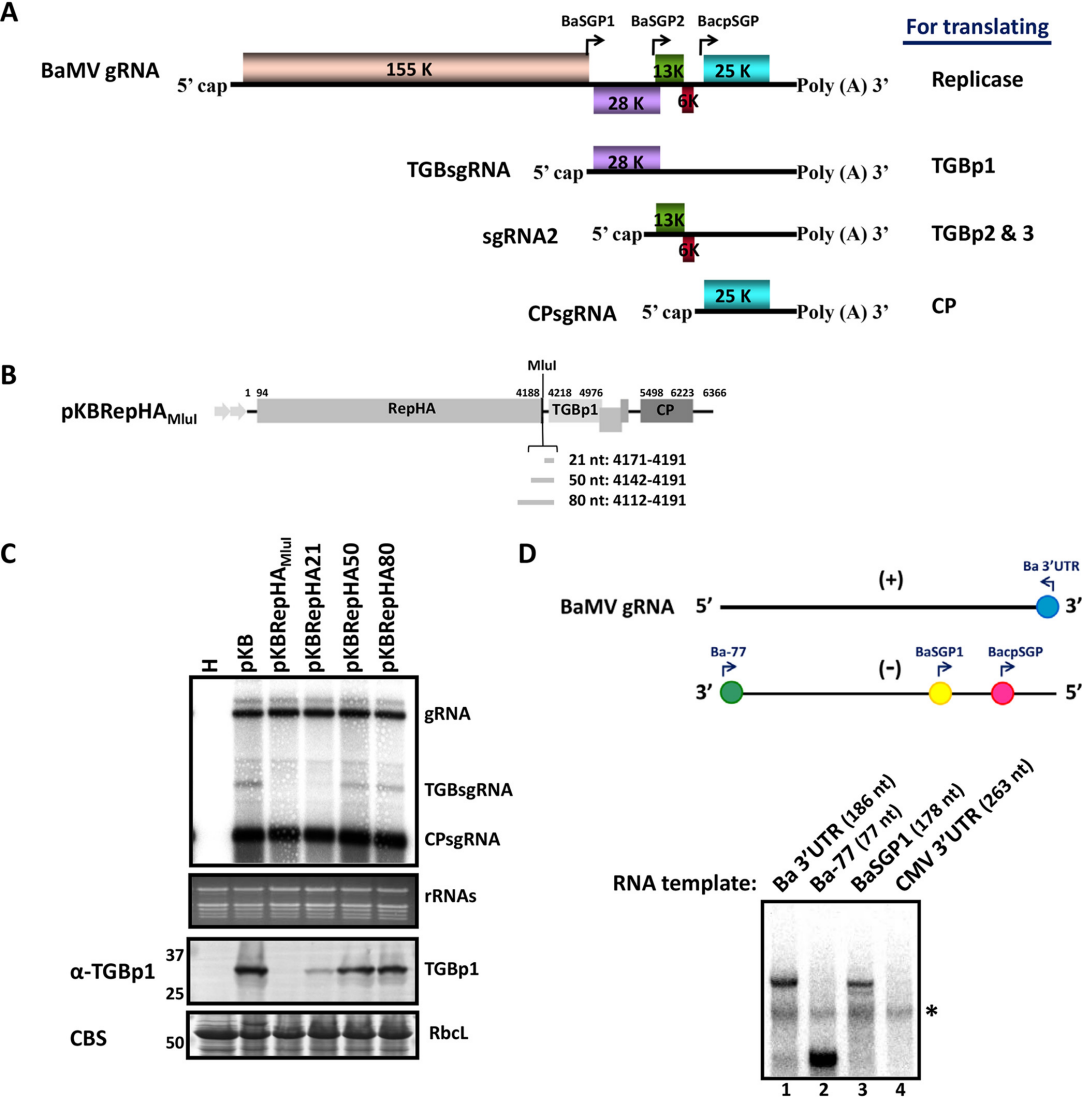
Mapping of the BaMV TGBp1 subgenomic RNA promoter. (A) Schematic diagrams of BaMV genomic RNA and subgenomic mRNAs. The 5′ and 3′ untranslated regions (UTRs) and viral open reading frames (boxes in different colors) are drawn according to scale. The translated proteins from each mRNA are indicated on the right. (B) Schematic representation of the genome organization of BaMV expressing RepHA (pKBRepHA_MluI_) driven by a dual 35S promoter (gray arrow). Five open reading frames of BaMV encode RepHA, TGBp1-3, and CP. The plasmid pKBRepHA_MluI_ was generated from pKB by insertion of the HA sequence and the MluI cut site at the 3′ end of the replicase coding region. The gray bars shown below indicate the 21-, 50-, and 80-nt insertions after the MluI site for restoring the putative promoter sequence for TGBp1 subgenomic RNA synthesis. (C) Northern blot analysis of the accumulation of BaMV genomic RNA (gRNA), subgenomic RNA for TGBp1 (TGBsgRNA), and subgenomic RNA for CP (CPsgRNA) in N. benthamiana plants individually infiltrated with pKB, pKBRepHA, pKBRepHA21, pKBRepHA50, and pKBRepHA80 for 3 days. Lane H represents a healthy plant without BaMV infection as a negative control. The ^32^P-labeled RNA probe for detecting BaMV RNAs was complementary to the 3′ end of the positive-strand RNA of BaMV. The ethidium bromide (EtBr)-stained gel shows rRNAs as the loading control. The lower panel shows protein analysis of the same set of samples. Total proteins were separated by 12% SDS-PAGE and stained with Coomassie blue (CBS) or probed with antiserum against BaMV TGBp1. Protein markers with molecular masses (in kDa) are shown on the left. RbcL, RuBisCO large subunit as the loading control. (D) Analysis of BaMV TGBsgRNA synthesized *in vitro* by BaMV RdRp preparation. The upper panel shows the schematic representation of the promoter locations of Ba 3′UTR, Ba-77, BaSGP1, and BacpSGP. RdRp assays were done with various transcripts, indicated above each lane, added exogenously as templates. The ^32^P-labeled products were analyzed by electrophoresis via a 5% native PAGE gel, followed by autoradiography. An asterisk indicates the nonspecific signal.

*Bamboo mosaic virus* (BaMV), belonging to the genus *Potexvirus* and family *Alphaflexiviridae*, has a single-stranded (+)RNA genome of approximately 6.4 kb with a 5′ cap structure and a 3′ poly(A) tail (12). The RNA genome is a multicistronic mRNA containing five ORFs (13, 14). ORF1 encodes a replicase (Rep) for BaMV replication (15). ORF2, −3, and −4 encode triple gene block protein 1 (TGBp1), TGBp2, and TGBp3, which are required for viral movement (16). TGBp1 is also an RNA silencing suppressor in BaMV infection (17). ORF5 encodes a capsid protein (CP) for virion assembly, movement, and symptom development (18–20). Like the strategy adopted for many (+)RNA viruses, BaMV produces three sgRNAs with 3′ co-termini to translate the downstream ORFs (Fig. 1A). Two major sgRNAs, namely, TGBsgRNA and CPsgRNA of approximately 2 and 1 kb, direct the translation of TGBp1 and CP, respectively (21), whereas sgRNA2, responsible for the translation of TGBp2 and TGBp3, accumulates in infected cells at a very low level.

The *cis*-acting elements, also known as promoter regions, for BaMV (−)RNA and (+)RNA synthesis have been identified in the 3′ untranslated region (3′UTR) of (+)RNA (Ba 3′UTR) and the 3′-terminal 77 nucleotides (nt) of (−)RNA (Ba-77), respectively (22, 23). Results from UV cross-linking assays indicated that the Ba 3′UTR interacts with several host factors that positively regulate BaMV replication, including chloroplast phosphoglycerate kinase (chlPGK), heat shock protein 90 (Hsp90), and glutathione *S*-transferase (GSTU4), whereas host factors that negatively regulate include elongation factor 1a (eEF1a) and glyceraldehyde 3-phosphate dehydrogenase (GAPDH) (24). The promoter region for the synthesis of CPsgRNA, spanning between nt −91 and +52 (the transcription start site [TSS] is designated +1) and which folds into four stem-loops (SLs) in the negative strand, has also been identified by using a BaMV satellite expression cassette (25). However, the identities and functions of promoter-interacting cellular proteins for regulating sgRNA synthesis remained elusive. Also, whether the same set of protein complexes was utilized in both the replication and transcription of viral RNAs required further analyses. Therefore, we sought to identify the host proteins interacting with promoter regions of both TGBsgRNA and CPsgRNA, namely, the subgenomic promoter-like sequences (SGPs) BaSGP1 and BacpSGP, respectively, and to further unveil the underlying mechanism for the regulation of sgRNA synthesis.

In this study, we have identified the Nicotiana benthamiana photosystem II (PSII) oxygen-evolving complex protein, NbPsbO1, in the partially purified replicase complexes extracted from BaMV-infected tissues and demonstrated that NbPsbO1 specifically interacted with both BaSGP1 and BacpSGP. Results of gene overexpression and silencing analyses revealed that NbPsbO1 is required for efficient transcription of BaMV sgRNAs but exerts no effect on gRNA replication. We further demonstrated that NbPsbO1 could be redistributed to chloroplast stroma, where the VRCs reside, following BaMV infection and may thus participate in the positive regulation of BaMV sgRNA synthesis. Together, our results unveiled a novel role of NbPsbO1 in BaMV infection cycles and provided further insights into the mechanism for differential regulation of BaMV gRNA and sgRNA synthesis.

## RESULTS

### Mapping of the BaMV TGBp1 sgRNA promoter *in vivo*.

In a previous study, by using the satBaMV expression cassette, BacpSGP has been mapped to span from nt −91 ∼ +52 relative to the transcription start site (TSS) (25). However, the location of BaSGP1 remained to be determined. Here, a BaMV infectious clone, pKBRepHA_MluI_, deficient in the transcription of TGBsgRNA, was used to map the promoter region for TGBsgRNA synthesis. The insertion of a hemagglutinin (HA) coding sequence upstream of the stop codon of the Rep gene in pKBRepHA_MluI_ disrupted an octanucleotide element, GUUAAGUA, representing conserved promoter-like sequences required for TGBsgRNA transcription among potexviruses (26, 27). To map BaSGP1, fragments of 21, 50, and 80 nt, conserving the octanucleotide element downstream of Rep gene, were inserted in pKBRepHA_MluI_, generating pKBRepHA21, pKBRepHA50, and pKBRepHA80, respectively, to test whether the transcription of TGBsgRNA could be restored (Fig. 1B). An inoculation assay revealed that in pKBRepHA50-infiltrated leaves, TGBsgRNA transcription was restored to the wild-type level at 3 days postagroinfiltration (dpai) (Fig. 1C, upper panel). Furthermore, consistent with RNA accumulation, the regenerated TGBsgRNA could serve as the template for translating TGBp1 protein (Fig. 1C, lower panel). Therefore, the region 65 nt upstream of the TSS of TGBsgRNA (25), at nt 4207 in the BaMV genome, is sufficient to direct transcription *in vivo*.

To validate the promoter region for recognition by BaMV RdRp complexes, an *in vitro* RdRp assay was performed with the minus strand (−) of nt −65 ∼ +113 relative to the TSS of TGBsgRNA, hereafter designated BaSGP1. In addition, Ba 3′UTR and Ba-77, containing promoter activities for initiation of (−) and (+) BaMV gRNA, respectively (23, 28) (Fig. 1D, upper panel), were used as positive controls in the *in vitro* RdRp assays. The result revealed that BaSGP1 could be recognized by the BaMV RdRp complexes as a template for synthesis with activity similar to that of Ba 3′UTR (Fig. 1D, compare lanes 3 and 1). In contrast, the 3′UTR containing the tRNA-like structure of cytomegalovirus (CMV) showed nondetectable template activity (Fig. 1D, lane 4), which demonstrated the template specificity of the BaMV RdRp preparation.

### Specific interaction of NbPsbO1 with the promoter regions for BaMV sgRNA synthesis.

**FIG 2:**
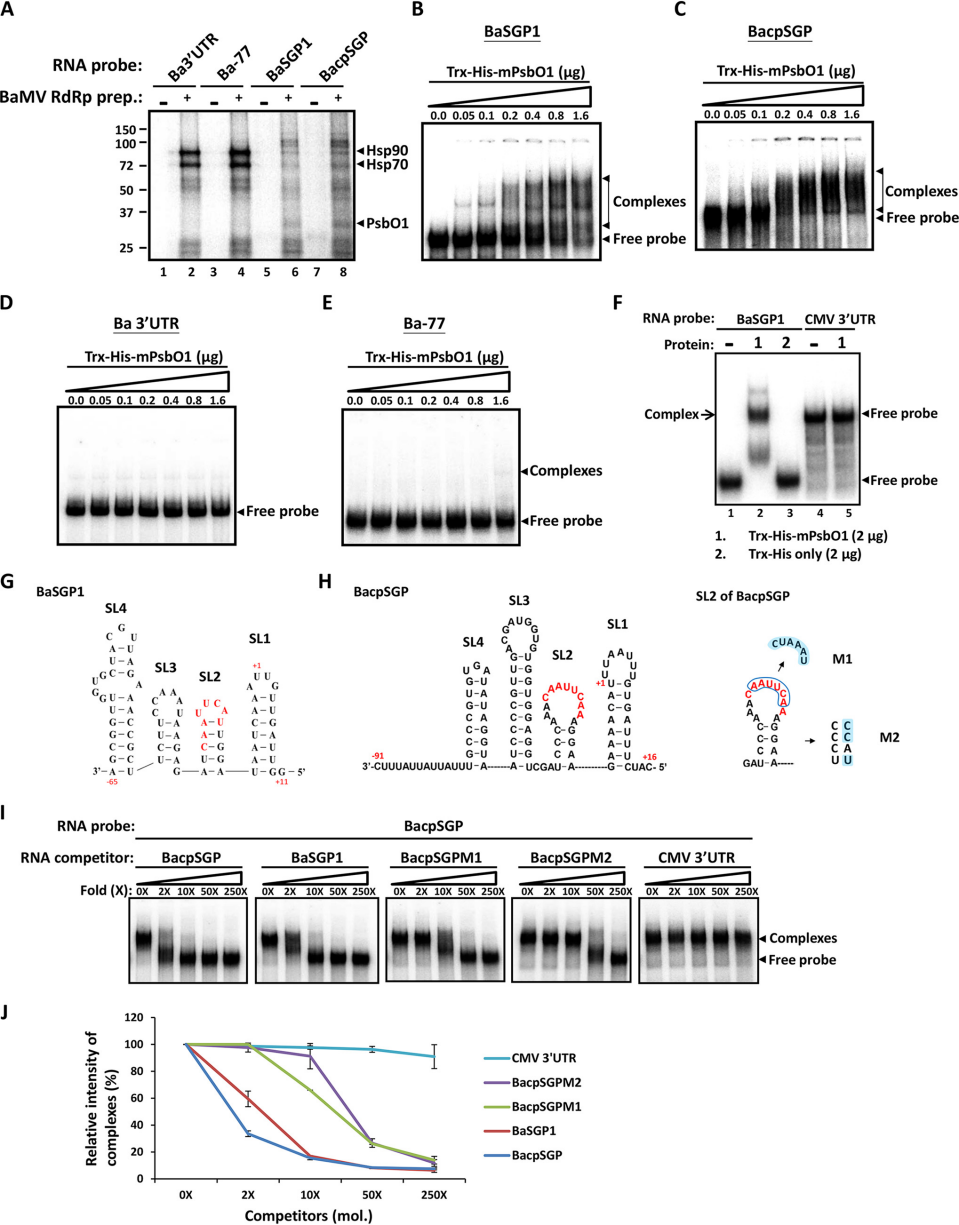
Identification of host factors that specifically interact with the promoter region for BaMV sgRNA synthesis in BaMV RdRp preparation. (A) Detection of host proteins interacting with BaMV 3′UTR, Ba-77, BaSGP1, or BacpSGP by UV cross-linking assay. The RNA probes were labeled with [α-^32^P]UTP and individually incubated with (+) or without (−) the BaMV RdRp preparation. The RNA-bound proteins were separated by electrophoresis on a 10% SDS-PAGE gel. The relative molecular weights of the proteins were estimated by the mobility in the gel in comparison with prestained markers, whose sizes (kDa) are indicated to the left of the panel. Arrowheads indicate positions of the candidate proteins, identified as Hsp90, Hsp70, and PsbO1 by mass spectrometry. (B to E) Interactions between the mature form of PsbO1 protein (Trx-His-mPsbO1) and various RNA probes tested with electrophoretic gel mobility shift assays (EMSAs). ^32^P-labeled BaSGP1, BacpSGP, Ba 3′UTR, and Ba-77 were incubated with increasing amounts of Trx-His-mPsbO1 (0, 0.05, 0.1, 0.2, 0.4, 0.8, and 1.6 μg). Reaction products were electrophoresed on a 5% native PAGE gel. The arrowheads indicate the positions of free probe and bound complex signals of corresponding RNA. (F) EMSA to examine the specific interaction between Trx-His-mPsbO1 and BaSGP1 RNA. Radiolabeled probes of BaSGP1 and CMV 3′UTR were incubated alone (lane −), with 2 μg Trx-His-mPsbO1 (lane 1), or with 2 μg Trx-His (lane 2) in a 10-μl reaction buffer for 15 min. Reaction products were electrophoresed on a 5% native PAGE gel. (G and H) Secondary structures of BaSGP1 and BacpSGP predicted by the program Mfold were converted to a linear format. The numbers are relative to the TSS of TGBsgRNA and CPsgRNA, respectively. The conserved octamer sequences are located in the loop and indicated in red. The boxed and shaded letters represent the mutated nucleotides in BacpSGPM1 (M1) and BacpSGPM2 (M2), respectively. (I) Characterization of binding activity between the BacpSGP and mPsbO1 by competitive EMSA. Trx-His-mPsbO1 (2 μg) was incubated with BacpSGP probe in the presence of 0-, 2-, 10-, 50-, and 250-fold molar excesses of unlabeled RNA competitors. (J) The relative intensity of each complex was quantified and is shown in a plot. The band intensity for binding without RNA competitor (0×) is defined as 100%. Data are the mean ± standard deviation (SD) of the results from three independent experiments.

To identify host proteins interacting with the promoter regions for BaMV sgRNA synthesis, we performed UV cross-linking assays to examine the binding of components in BaMV RdRp preparations with Ba 3′UTR, Ba-77, BaSGP1, and BacpSGP. In this study, BacpSGP refers to the minus strand of nt −91 ∼ +135 relative to the TSS of CPsgRNA, containing CP sgRNA promoter (25). Several differential binding signals were detected in BaMV RdRp preparations with promoters for BaMV gRNA and sgRNA synthesis (Fig. 2A). Hsp90 and Hsp70, previously found to bind to Ba 3′UTR and Ba-77 and assist in the assembly of BaMV replication complexes (29, 30), showed high affinity for Ba 3′UTR and Ba-77 (Fig. 2A, lanes 2 and 4). In contrast, the binding affinities of Hsp90 and Hsp70 to BaSGP1 and BacpSGP were much lower (Fig. 2A, lanes 6 and 8). A protein with an estimated molecular weight of about 30 kDa in BaMV RdRp complexes exhibited specific binding to BaSGP1 and BacpSGP (Fig. 2A, lanes 6 and 8). Following analysis by matrix-assisted laser desorption ionization–time of flight mass spectrometry (MALDI-TOF MS), the 30-kDa protein was identified as the chloroplast PsbO1 protein, one of three extrinsic protein subunits of the PSII supercomplex (31). PsbO1 is nucleus encoded, and the premature protein contains a chloroplast-targeting peptide (CTP) and a thylakoid signal peptide (TSP), which are both cleaved to generate mature protein (mPsbO1) after successfully targeting the thylakoid lumen (32). To test whether NbPsbO1 interacts directly with BaMV SGPs, we performed an electrophoretic mobility shift assay (EMSA) with recombinant NbPsbO1 expressed from Escherichia coli. A previous study showed that fusion of spinach PsbO to thioredoxin (Trx) had a crucial impact on the solubility of the recombinant PsbO (33). Therefore, we designed primers to amplify and clone the full-length NbPsbO1 coding region into pET32a to express in E. coli as a thioredoxin-His_6_ fusion protein, Trx-His-PsbO1. However, the recombinant premature form of NbPsbO1 was still expressed as an insoluble protein and sometimes was degraded. We then tried to produce the mature form of NbPsbO1, Trx-His-mPsbO1, which was efficiently expressed with high solubility that allowed for the purification of Trx-His-mPsbO1 under native conditions in E. coli. Following EMSA, mobility shift signals were observed when BaSGP1 and BacpSGP probes were incubated with 50 ng of Trx-His-mPsbO1, and the extent of the mobility shift increased with an increase in concentration (100 ng to 1.6 μg) of Trx-His-mPsbO1 (Fig. 2B and C). In contrast, no obvious RNA-protein complex was observed when 1.6 μg of Trx-His-mPsbO1 was added to the riboprobes of Ba 3′UTR or Ba-77 (Fig. 2D and E). The Trx-His tag alone did not interact with BaSGP1 (Fig. 2F, lane 3), which confirms that mPsbO1 is responsible for the binding of Trx-His-mPsbO1 to BaSGP1 and BacpSGP. These results demonstrated the specificity of mPsbO1 binding to the promoter regions for BaMV sgRNA synthesis but not those for gRNA synthesis. In addition, Trx-His-mPsbO1 did not interact with the CMV 3′UTR (Fig. 2F, lane 5), which further verified that the mPsbO1 binding is specific to BaMV SGPs. To elucidate whether the binding of NbPsbO1 to the BaMV SGPs is functionally relevant to BaMV sgRNA synthesis, competitive EMSAs were performed with two BacpSGP mutant competitors, BaSGPM1 and BaSGPM2, in which the loop sequence was altered or the structure of BacpSGP SL2 was disrupted, respectively (Fig. 2H, right panel). Both mutants have been shown to suppress the activity of BacpSGP for transcription (25). The results showed that BacpSGPM1 and BacpSGPM2 did not efficiently outcompete BacpSGP for binding with mPsbO1 when compared to the competition efficiency of unlabeled BacpSGP (Fig. 2I and J). This observation revealed that NbPsbO1 preferentially interacts with functional BacpSGP and suggested that interference of the binding between BacpSGP and NbPsbO1 may decrease the BacpSGP activity. Thus, the binding of NbPsbO1 to BaMV SGPs may be involved in the regulation of BaMV sgRNA transcription.

### Knocking down *NbPsbO1* reduced accumulation of BaMV RNA in N. benthamiana plants and protoplasts.

**FIG 3:**
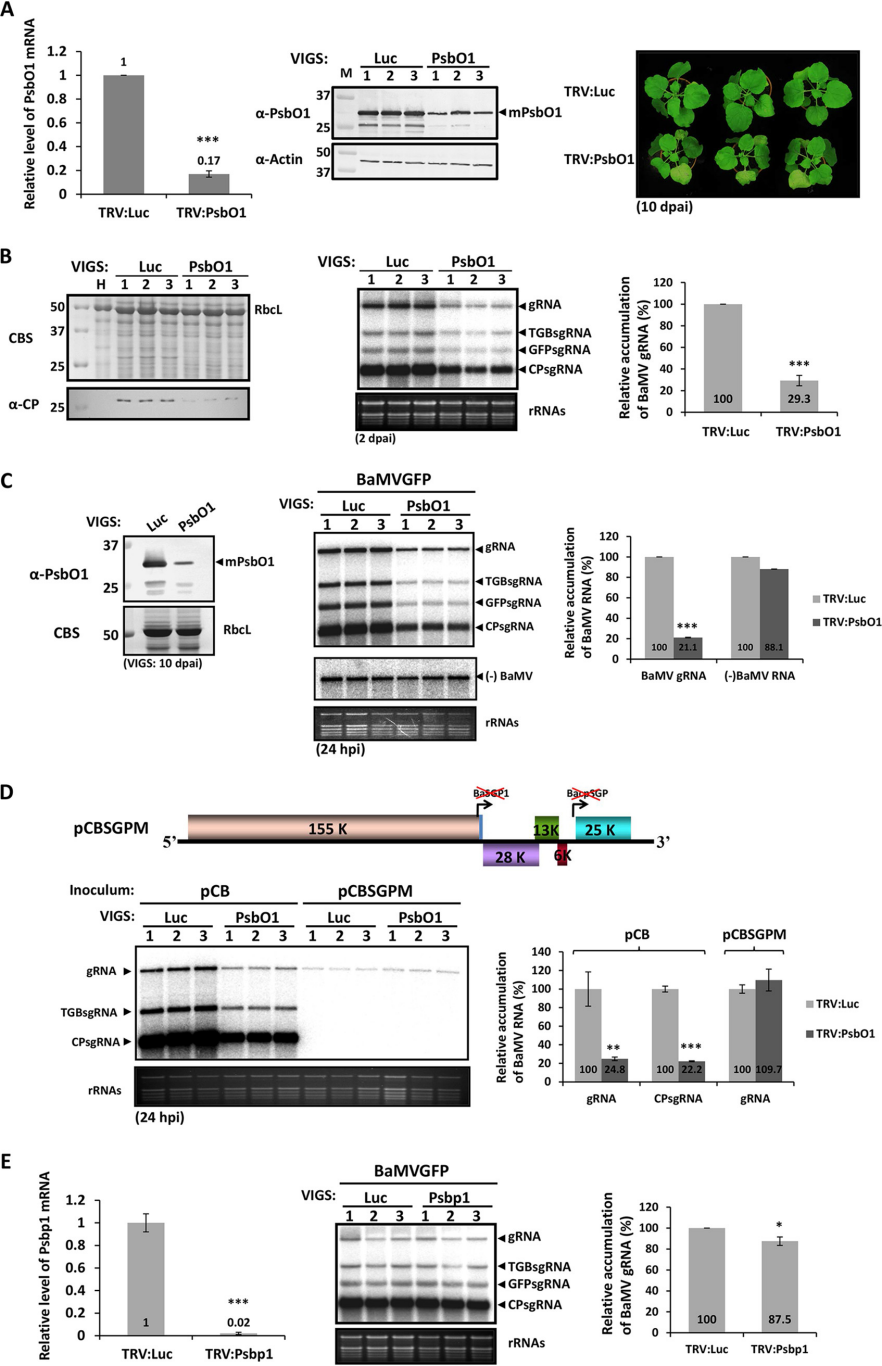
Effect of *NbPsbO1* knockdown on BaMV accumulation in N. benthamiana plants and protoplasts. (A) Characterization of *NbPsbO1*-knockdown N. benthamiana plants. The TRV-based VIGS system was used to silence the luciferase gene (*Luc*) and *NbPsbO1*. The relative expression of *NbPsbO1* in control (TRV:Luc) and *PsbO1*-knockdown (TRV:PsbO1) N. benthamiana plants was measured by quantitative reverse-transcription PCR (RT-qPCR) at 10 days post-agroinfiltration (dpai). The middle panel shows the protein analysis of the same set of samples. Total proteins were separated by 12% SDS-PAGE and stained with Coomassie blue or probed with antiserum against PsbO1 and actin. The right panel shows the phenotype of *PsbO1*-knockdown N. benthamiana. (B) Western and Northern blot analyses of the levels of BaMV in VIGS-mediated *PsbO1*-knockdown plants. At 10 dpai, the upper leaves of control or silenced plants were infiltrated with pKBG. Infiltrated leaves were tested at 2 dpai for BaMV accumulation by Western (left panel) and Northern (middle panel) blot analyses with antiserum against BaMV CP and ^32^P-labeled RNA probe for detecting BaMV CP and RNAs, respectively. The right panel shows the relative accumulation of BaMV gRNA derived from the left panel. (C) (+) and (−) BaMV RNA accumulation in control and *NbPsbO1*-knockdown protoplasts. The right panel shows the relative accumulation of BaMV gRNA and (−)BaMV RNA derived from the left panel. (D) Northern blot analyses of levels of BaMV gRNA and sgRNAs in control and *NbPsbO1*-knockdown protoplasts inoculated with pCB or pCBSGPM at 24 h postinfection (hpi). The upper panel is a schematic diagram of a BaMV SGP inactive infectious clone, pCBSGPM. The right panel shows the relative accumulation of BaMV gRNA and CPsgRNA derived from the left panel. (E) Relative accumulation of BaMV gRNA in *NbPsbp1*-knockdown plants. The left panel shows the NbPsbp1 level in *NbPsbp1*-knockdown (TRV:PsbP1) and negative-control (TRV:Luc) plants, as determined by RT-qPCR. The accumulation of BaMV RNA was assayed by Northern blot analysis. Data are means ± SDs from three independent experiments. Asterisks indicate statistically significant differences between the indicated groups by Student’s *t* test (*, *P* < 0.05; **, *P* < 0.01; ***, *P* < 0.001).

To investigate the involvement of NbPsbO1 in BaMV infectivity, we downregulated the expression of *NbPsbO1* by virus-induced gene silencing (VIGS). Four isoforms of *NbPsbO* were reported to show high identity (34) and thus might play a redundant role in BaMV infection. To avoid the gene redundancy problem, a 500-bp fragment from the coding sequence of *NbPsbO1* highly conserved among the corresponding regions of other isoforms was cloned into a TRV-based silencing vector (35) to generate TRV:PsbO1, which was cointroduced into N. benthamiana with TRV1 by *Agrobacterium*-mediated infiltration. *NbPsbO1* expression was decreased to 17% of that of mock-infiltrated plants (TRV:luciferase [TRV:Luc]) at 10 days post-agroinfiltration (dpai) (Fig. 3A, left panel). However, downregulation of *NbPsbO* expression in N. benthamiana resulted in a yellowing phenotype (Fig. 3A, right panel) that should result from decreased leaf concentrations of photosynthetic pigments, chlorophylls and carotenoids (36). To investigate the impact of *NbPsbO* downregulation on BaMV accumulation, upper leaves from *Luc*- and *NbPsbO*-silenced N. benthamiana plants were agroinfiltrated with pKBG, an infectious clone of a recombinant BaMV, BaMVGFP, which harbors the coding sequences of green fluorescent protein (GFP) (37). The accumulation of viral gRNA in BaMVGFP-infected *NbPsbO*-knockdown plants was reduced to approximately 29.3% of that in control plants (Fig. 3B). Thus, the result suggested that NbPsbO1 plays a positive role in promoting BaMV accumulation. To investigate how NbpsbO1 affects the accumulation of BaMV in N. benthamiana, protoplasts derived from the *NbPsbO1*-knockdown plants were prepared and inoculated with viral RNA of BaMVGFP. The accumulation of BaMV gRNA was decreased to 21% of that of control protoplasts at 24 h postinoculation, but the accumulation of minus-strand RNA was not affected (Fig. 3C). These results of RNA accumulation analysis in protoplasts indicated that NbPsbO1 functions mainly in BaMV (+)RNA accumulation instead of viral movement. To further elucidate whether NbPsbO1 targets only the SGPs for sgRNA transcription, a BaMV mutant with inactivated SGPs, designated BaMV-SGPM, was used to test the effect of NbPsbO1 on viral gRNA replication independent from sgRNA transcription. The BaSGP1 and BacpSGP in pCBSGPM were inactivated by the insertion of HA in place of the conserved octamer motif downstream of the replicase ORF and the disruption of the SL2 structure, respectively (Fig. 3D). The result of the inoculation assay showed that there was no significant difference between the accumulation levels of BaMV-SGPM gRNA in protoplasts prepared from *NbPsbO1*-knockdown or control N. benthamiana plants (Fig. 3D), indicating that NbPsbO1 functions mainly in sgRNA synthesis without affecting gRNA replication. The decrease in gRNA accumulation in BaMVGFP-infected *NbPsbO*-knockdown plants (Fig. 3B) might have resulted from the decrease in CP accumulation (Fig. 3B), which may interfere with virion formation and thereby impair the protection of gRNA from the host degradation system. NbPsbO is one of the oxygen-evolving complex (OEC) protein subunits involved in photosynthesis which play vital roles in virus infection (38). To distinguish between the specific effect of NbPsbO1 on BaMV replicase complex and the pleiotropic effect on photosynthesis machinery that affects BaMV RNA accumulation in plants, a gene knockdown experiment was performed using another component of the OEC, NbPsbP1, as follows. A 400-bp fragment representing partial NbPsbP1 coding sequence (CDS) was inserted into the TRV-based VIGS vector and used for silencing the expression of *NbPsbP1* in N. benthamiana. The expression of *NbPsbP1* in knockdown plants was reduced to 2% of that of the control (Fig. 3E, left panel), and the accumulation of BaMV RNA in *NbPsbp1*-knockdown leaves was slightly reduced, to 88% of that of control (Fig. 3E, right panel). NbPsbp1 did not significantly affect BaMV infection, indicating that the reduction in BaMV accumulation by knockdown of *NbPsbO1* was not mainly due to the pleiotropic effects or disrupting the chloroplast.

### Overexpression of NbPsbO1 increased BaMV sgRNA accumulation.

**FIG 4:**
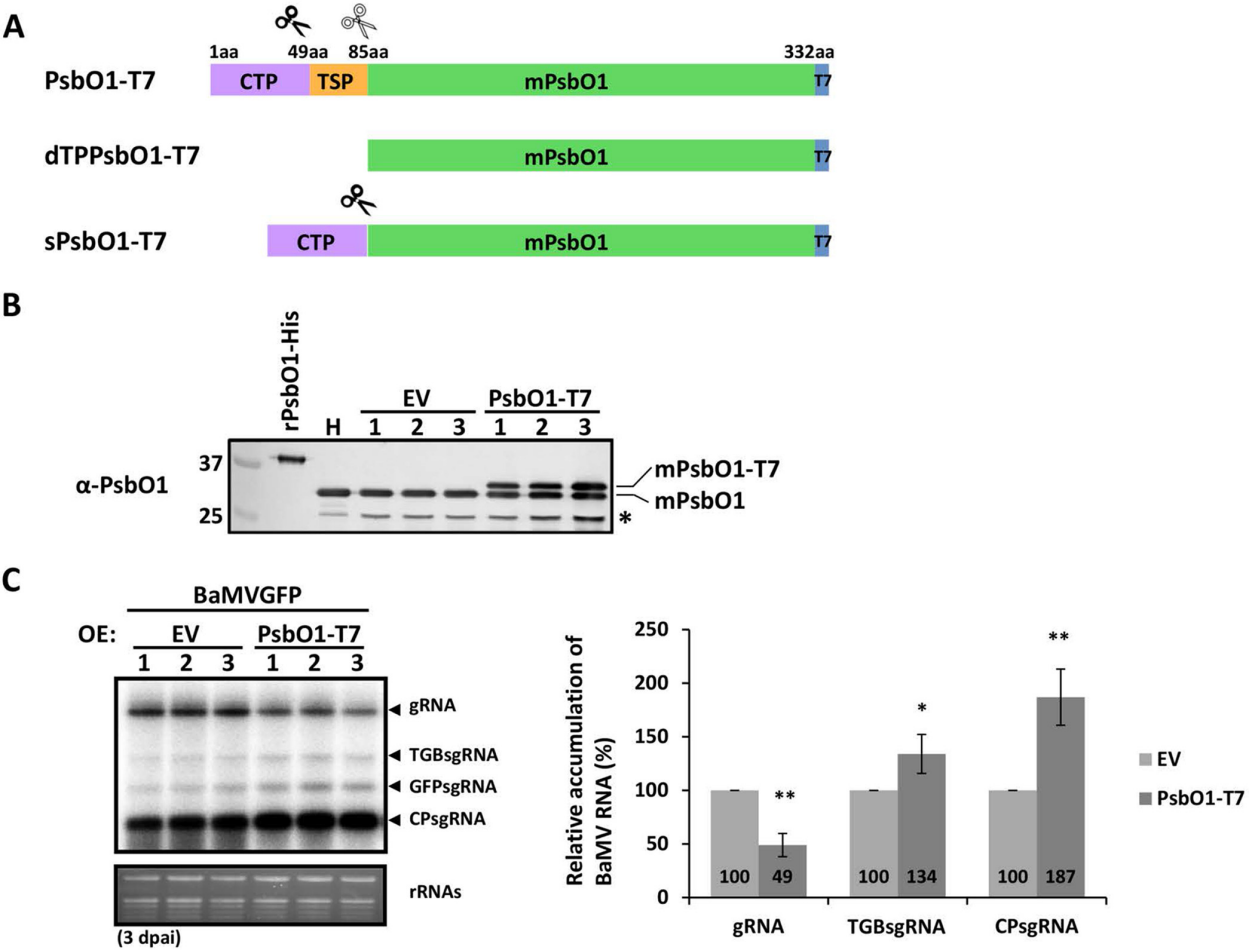
Effect of transiently overexpressed NbPsbO1 on BaMV accumulation. (A) Schematic diagram of the full-length NbPsbO1 and its derivatives with a C-terminal tag, T7. Chloroplast transit peptide (CTP) is a cleavable signal that targets protein from the cytosol to the chloroplast stroma, thylakoid signal peptide (TSP) is a cleavable signal that targets protein from the chloroplast stroma to the thylakoid lumen, and mPsbO1 is the mature form of NbPsbO1. (B) Immunoblot analysis of transiently expressed empty vector (EV) or PsbO1-T7 at 3 dpai by *Agrobacterium*-mediated expression in N. benthamiana. Total proteins were separated on a 12% SDS-PAGE gel, and anti-PsbO1 antibody was used to detect endogenous mPsbO1 and transiently expressed mPsbO1-T7. *, degraded form of PsbO1. rPsbO1-His, purified recombinant PsbO1-His as a molecular weight marker. (C) Northern blot analyses of accumulation of BaMVGFP RNA in N. benthamiana transiently overexpressing (OE) EV or PsbO1-T7. Total RNA was isolated at 3 dpai. EtBr-stained rRNA is shown to demonstrate equal loading. The right panel shows the quantification of the relative accumulation of BaMV gRNA, TGBsgRNA, and CPsgRNA, derived from the left panel. Data are the mean ± SD of the results from at least three experiments (Student's *t* test: *, *P* < 0.05; **, *P* < 0.01).

To corroborate the positive role of NbPsbO1 in BaMV accumulation, we performed a functional analysis by transiently overexpressing NbPsbO1. Full-length NbPsbO1 coding sequence with a T7 tag fusion at the C terminus was amplified and cloned into the expression vector to generate pBINbPsbO1-T7 (Fig. 4A). The role of NbPsbO1 in BaMV accumulation was validated by analysis of BaMV RNA accumulation in N. benthamiana leaves coinfiltrated with NbPsbO1-T7 and BaMVGFP at 3 dpai. The ectopically expressed NbPsbO1-T7 was detected by Western blot analysis with antiserum against NbPsbO1 as the protein band with higher molecular weight than that of the endogenous NbPsbO1 (Fig. 4B). The CTP and TSP of both endogenous and ectopically expressed NbPsbO1 were cleaved in N. benthamiana to generate the mature forms of PsbO1 (mPsbO1) and PsbO1-T7 (mPsbO1-T7), respectively, with lower molecular weights than that of the full-length rPsbO1-His purified from E. coli (Fig. 4B). Northern blot analyses showed that the accumulation of TGBsgRNA and CPsgRNA was 1.34- and 1.87-fold greater, respectively, with overexpression of NbPsbO1-T7 than that in control plants (infiltrated with empty vector [EV]) (Fig. 4C). The observation indicated that NbPsbO1 promotes BaMV sgRNA transcription. However, the accumulation of BaMV gRNA in NbPsbO1-T7 overexpressed plants was decreased to 49% of that in control plants (Fig. 4C), which implies that overexpression of NbPsbO1 led to excessive transcription of BaMV sgRNA, which might compete with the synthesis of BaMV gRNA for (−)RNA template.

### BaMV infection altered the subcellular localization of NbPsbO1.

**FIG 5:**
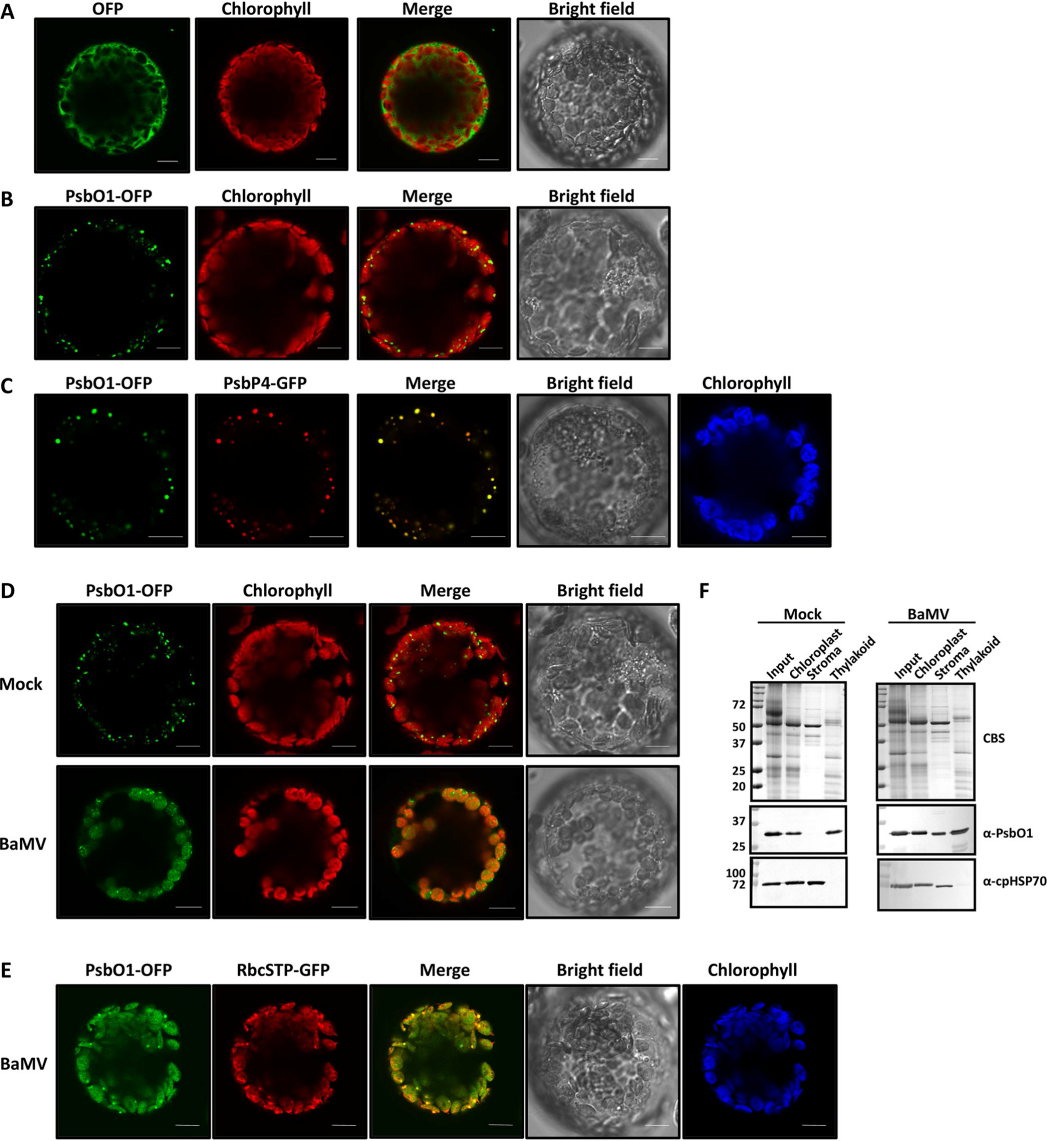
Subcellular localization of PsbO1 in N. benthamiana protoplasts. OFP (A), PsbO1 C terminus fused with OFP (PsbO1-OFP) (B), and PsbO1-OFP along with PsbP4-GFP (C) were transiently expressed in N. benthamiana. The protoplasts were prepared at 3 dpai and examined by confocal microscopy. (D) Protoplast cells were isolated from leaves expressing PsbO1-OFP along with mock or BaMV infection as indicated. (E) Protoplast cells were isolated from BaMV-infected leaves that coexpressed PsbO1-OFP and RbcSTP-GFP. The OFP signal is artificially colored green. The autofluorescence of chloroplasts (chlorophyll) is red in panels A, B, and D and blue in panels C and E. Bar, 10 μm. (F) Separation of chloroplasts isolated from mock-infected (left) or BaMV-infected (right) leaves into fractions of stroma and thylakoid, followed by immunodetection of PsbO1 proteins. Coomassie blue staining shows protein profiles of chloroplast fractions. The chloroplastic form of Hsp70 (cpHSP70) was detected as a marker of the stroma fraction.

To examine the subcellular localization of NbPsbO1, we constructed wild-type NbPsbO1 with a C-terminal fusion of orange fluorescent protein (OFP), designated NbPsbO1-OFP, for transient expression in N. benthamiana. In contrast to the distribution of OFP in the cytoplasm (Fig. 5A), the subcellular localization of NbPsbO1-OFP in N. benthamiana protoplasts was observed in chloroplasts as punctate structures (Fig. 5B), which suggested that NbPsbO1-OFP, along with NbPsbP and NbPsbQ, might have formed OEC-like structures in the thylakoid membrane-lumen interface (39). To test this hypothesis, NbPsbO1-OFP and an OEC marker, NbPsbP-GFP, were coexpressed in protoplasts and examined by confocal microscopy, and the results showed colocalization of both proteins in the punctate structure (Fig. 5C), which suggested that ectopically expressed NbPsbO1 forms OEC with NbPsbP in the thylakoid. To determine the effect of BaMV infection on the localization pattern of NbPsbO1, we expressed NbPsbO1-OFP in mock- or BaMV-infected N. benthamiana (Fig. 5D). At 3 dpai of BaMV, the expression signal of NbPsbO1-OFP was altered from the OEC-like punctate structure to a stroma-diffused pattern (Fig. 5D). To confirm the localization of NbPsbO1 after BaMV infection, we coexpressed NbPsbO1-OFP with a chloroplast stroma marker, a transient peptide of ribulose-1,5-bisphosphate carboxylase/oxygenase (RuBisCO) S subunit with a C-terminal fusion of GFP (RbcSTP-GFP) (40). The result of confocal microscopic examination showed an overlap of OFP and GFP in BaMV-infected protoplast cells (Fig. 5E). Thus, BaMV infection can alter the suborganelle localization of NbPsbO1 from the thylakoid membrane to chloroplast stroma. To clearly determine the subcellular localization of NbPsbO1 during BaMV infection, a fractionation assay was performed to isolate chloroplast subcompartments, stroma and thylakoid, for analysis of NbPsbO1 localization by immunoblotting. The results revealed that PsbO1 was detected mainly in the thylakoid while some PsbO1 was translocated into stroma in the BaMV-infected cells (Fig. 5F), consistent with the observation of NbPsbO1 distribution during BaMV infection (Fig. 5D).

### Overexpression of an NbPsbO1 mutant with CTP and TSP deletion negatively regulated BaMV accumulation in N. benthamiana.

**FIG 6:**
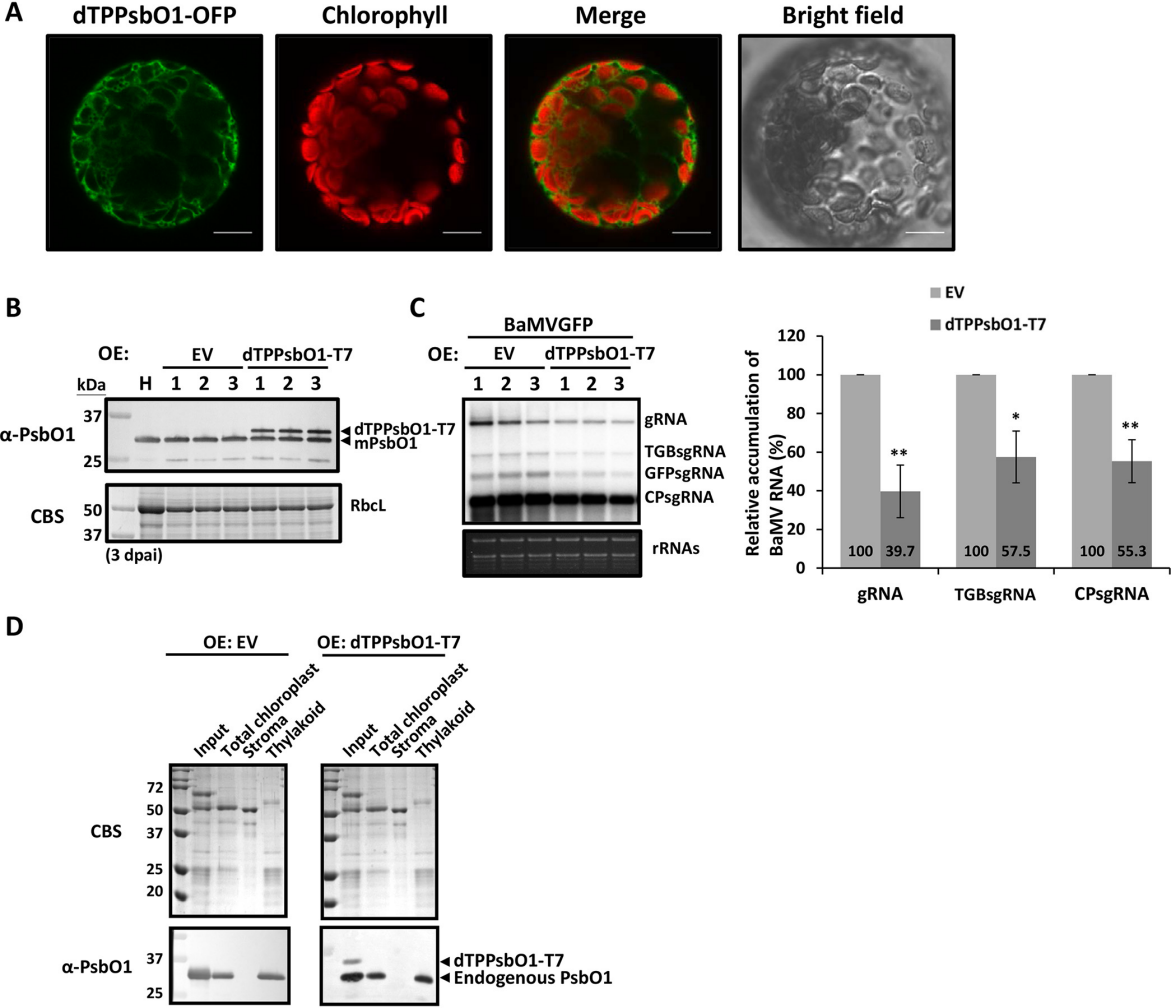
Effect of mislocalized NbPsbO1 on BaMV accumulation. (A) Subcellular localization of dTPPsbO1-OFP in N. benthamiana protoplasts. Protoplast cells were isolated from leaves expressing dTPPsbO1-OFP at 3 dpai. The signal for OFP is green, and that for chlorophyll is red. Scale bars, 10 μm. (B) Immunoblot analysis of transiently expressed EV or dTPPsbO1-T7 at 3 dpai in N. benthamiana. Total proteins were separated on a 12% SDS-PAGE gel, and anti-PsbO1 antibody was used to detect endogenous mPsbO1 and transiently expressed dTPPsbO1-T7. (C) Northern blot analyses of accumulation of BaMVGFP RNA in N. benthamiana transiently overexpressing EV or dTPPsbO1-T7. Total RNA was isolated at 3 dpai. EtBr-stained rRNA is shown for equal loading. The right panel shows the quantification of the relative accumulation of BaMV gRNA, TGBsgRNA, and CPsgRNA, derived from the left panel. Data are the mean ± SD of the results from at least three experiments (Student's *t* test: *, *P* < 0.05; **, *P* < 0.01). (D) Detection of endogenous PsbO1 in chloroplasts and their subcompartments purified from the leaves expressing EV or dTPPsbO1-T7. The Percoll-purified chloroplast and subplastidial fractions (stroma and thylakoid) were separated by electrophoresis on a 12% SDS-PAGE gel and stained with Coomassie blue or detected endogenous PsbO1 and transiently expressed dTPPsbO1-T7 by anti-PsbO1 antibody.

To test whether the chloroplast importation of NbPsbO1 is important for efficient BaMV accumulation, we generated a mutant with a deletion of both transit peptides, CTP and TSP, and a C-terminal fusion of OFP (dTPPsbO1-OFP) and performed the following experiments. First, the dTPPsbO1-OFP was expressed in protoplasts and examined by confocal microscopy, which revealed its subcellular localization in the cytoplasm, demonstrating the requirement of the functional transit peptides for import of NbPsbO1 into the chloroplast thylakoid (Fig. 6A). To understand the effect of NbPsbO1 subcellular localization on BaMV infection, the construct of dTPPsbO1-T7, without CTP and TSP (Fig. 4A), was coinfiltrated with pKBG in *N. bemthamiana*. At 3 dpai, the protein accumulation of endogenous mPsbO1 and exogenous dTPPsbO1-T7 was verified by immunoblotting (Fig. 6B). BaMV RNA accumulation was significantly lower in plants transiently expressing dTPPsbO1-T7 than in control plants (Fig. 6C). Thus, the result indicated that proper localization of NbPsbO1 into chloroplasts is required for efficient accumulation of BaMV in N. benthamiana. The mislocalized NbPsbO1 may have played a dominant negative role on BaMV infection, diminishing the functions of the endogenous NbPsbO1. To investigate whether the expression of mislocalized NbPsbO1 affects or interferes with the location or accumulation of the endogenous NbPsbO1, the chloroplast subcompartments were isolated from leaves infiltrated with empty vector (EV) or the construct expressing dTPPsbO1-T7, and localization of PsbO1 was detected by immunoblotting. The results showed that neither the accumulation nor location of the endogenous NbPsbO1 protein was affected by dTPPsbO1-T7 (Fig. 6D). This observation raised the possibility that dTPPsbO1 may have sequestered other proviral host factors for BaMV transcription in the cytoplasm and thus led to interference with BaMV accumulation.

### Overexpression of an NbPsbO1 mutant with a TSP deletion increased BaMV sgRNA accumulation in N. benthamiana.

**FIG 7:**
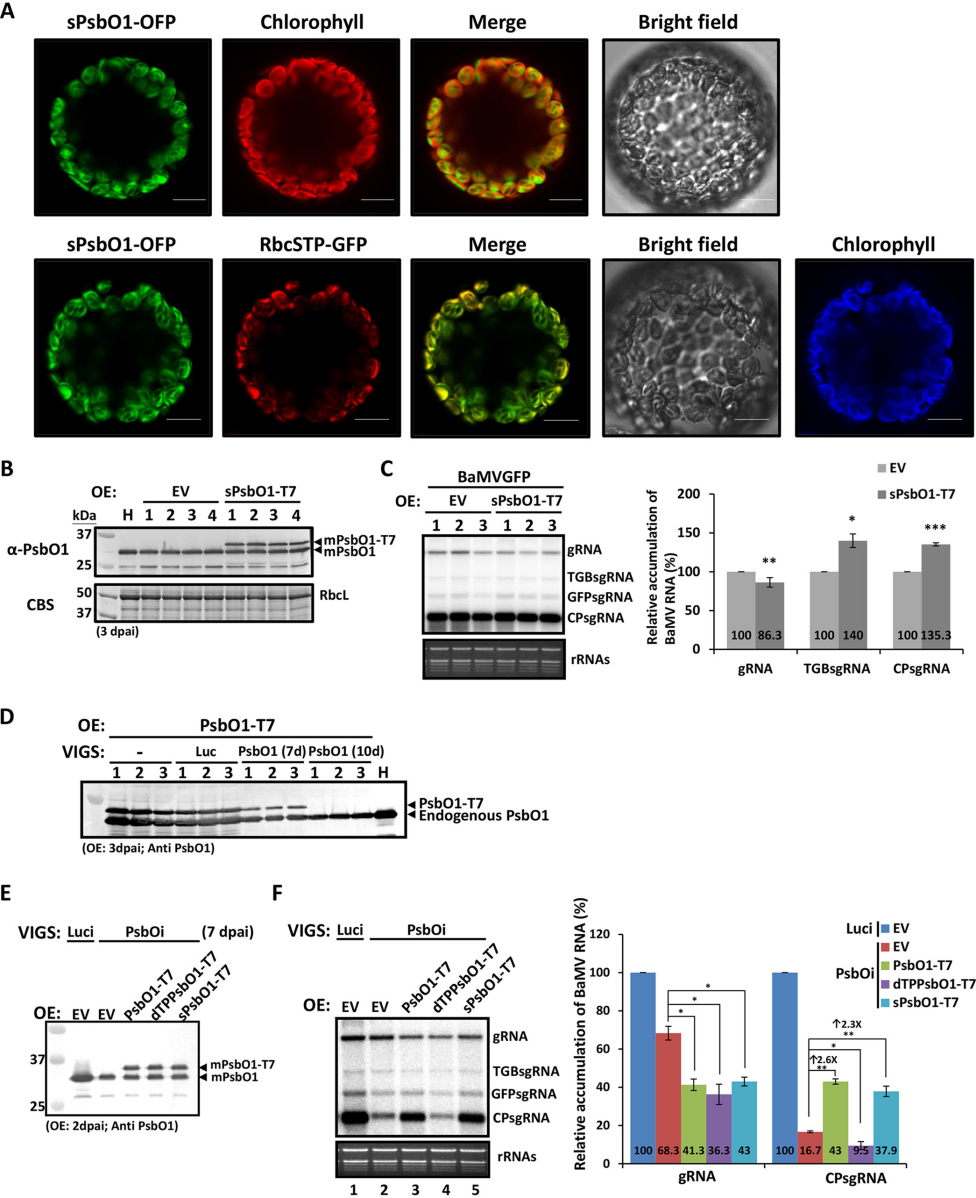
Effect of transiently overexpressed NbPsbO1 derivative on BaMV accumulation. (A) Subcellular localization of sPsbO1-OFP in N. benthamiana protoplasts. Protoplast cells were isolated from leaves expressing sPsbO1-OFP (upper panels) or sPsbO1-OFP along with RbcSTP-GFP (lower panels) at 3 dpai. Scale bars, 10 μm. (B) Immunoblot analysis of transiently expressed EV or sPsbO1-T7 at 3 dpai in N. benthamiana. Total proteins were separated on a 12% SDS-PAGE gel, and anti-PsbO1 antibody was used to detect endogenous mPsbO1 and transiently expressed mPsbO1-T7. (C) Northern blot analyses of accumulation of BaMVGFP RNA in N. benthamiana transiently overexpressing EV or sPsbO1-T7. Total RNA was isolated at 3 dpai. The right panel shows the quantification of the relative accumulation of BaMV gRNA, TGBsgRNA, and CPsgRNA, derived from the left panel. Data are the mean ± SD of the results from at least three experiments (Student's *t* test: *, *P* < 0.05; **, *P* < 0.01; ***, *P* < 0.001). (D) Detection of overexpressed PsbO1-T7 in *PsbO1*-silenced plants. Immunoblot analysis of transiently expressed PsbO1-T7 at 3 dpai in healthy (−) or VIGS of *Luc-* or *PsbO1-*silenced N. benthamiana plants. Total proteins were separated on a 12% SDS-PAGE gel, and anti-PsbO1 antibody was used to detect endogenous PsbO1 and transiently expressed PsbO1-T7. 7d and 10d indicate PsbO1 expressed at 7 or 10 days after *PsbO1* was silenced. (E) Immunoblot analysis of transiently expressed EV and NbPsbO1 derivatives at 2 dpai in *Luc*-knockdown (Luci) or *PsbO1*-knockdown (PsbOi) N. benthamiana leaves. The plants were silenced for 7 days and then coinfiltrated with pKBG and the corresponding clone as indicated above each lane. (F) Northern blot analyses of accumulation of BaMVGFP RNA in Luci or PsbOi N. benthamiana transiently overexpressing EV or NbPsbO1 derivatives. Total RNA was isolated at 2 dpai. The right panel shows the quantification of the relative accumulation of BaMV gRNA and CPsgRNA, derived from the left panel. Data are the mean ± SD of the results from at least three experiments (Student's *t* test: **, *P* < 0.01).

The above results suggested that proper chloroplast localization of NbPsbO1 is important for BaMV accumulation, and BaMV infection can alter the subcellular localization of NbPsbO1 to chloroplast stroma. We speculated that the stroma localization of NbPsbO1 may promote the accumulation of BaMV gRNA and/or sgRNAs. To investigate this hypothesis, we generated a TSP deletion mutant to examine the effect of stroma-localized NbPsbO1 (sPsbO1) on BaMV accumulation. The results of confocal microscopy examination showed colocalization of sPsbO1-OFP and the stroma marker RbcSTP-GFP, demonstrating the chloroplast stroma localization of sPsbO1 in N. benthamiana protoplasts (Fig. 7A). We then coinfiltrated the sPsbO1-T7 construct (Fig. 4A) and pKBG into N. benthamiana. At 3 dpai, protein accumulation of endogenous mPsbO1 and exogenous mPsbO1-T7 was verified by immunoblotting (Fig. 7B). BaMV sgRNA accumulation was increased by about 1.4-fold in plants transiently expressing sPsbO1-T7 compared to that in control plants (Fig. 7C). Thus, the localization of NbPsbO1 into chloroplast stroma enhanced the efficient accumulation of BaMV sgRNA in N. benthamiana. However, the enhancement was not quite remarkable. To magnify the effects of ectopically expressed NbPsbO1 on BaMV accumulation, PsbO1-T7 and its derivatives were coexpressed with BaMV-GFP in the *NbPsbO1*-silenced plants at 7 days postinfiltration of the silencing-inducing construct TRV:PsbO1, at which stage the silencing efficiency is lower to prevent *PsbO1* mRNAs from being totally degraded by the ongoing RNA silencing (Fig. 7D). The transiently expressed PsbO1-T7 and its derivatives in *NbPsbO1*-silenced plants (PsbOi) were verified by immunoblotting (Fig. 7E). The accumulation of BaMV gRNA and sgRNA in PsbOi plants was decreased to 68.3% and 16.7% of that in control plants (Luci), respectively (Fig. 7F, compare lanes 2 and 1). Notably, in PsbOi plants, BaMV CPsgRNA accumulation was 2.6- and 2.3-fold greater with overexpression of PsbO1-T7 and sPsbO1-T7, respectively, than that in control plants (infiltrated with EV) (Fig. 7F, compare lanes 3 and 5 to lane 2). Overexpression of dTPPsbO1-T7 in PsbOi plants negatively affected BaMV RNA accumulation (Fig. 7F, compare lanes 4 and 2), which is consistent with the above observations (Fig. 6C). Thus, the ectopically expressed wild-type PsbO1-T7 and sPsbO1-T7 could compensate for the function of silenced *NbPsbO1* to promote BaMV sgRNA synthesis, which suggested that the stroma localization of NbPsbO1 contributes to the enhancement of BaMV sgRNA synthesis.

### Depletion of NbPsbO1 reduced BaMV sgRNA synthesis in an *in vitro* RdRp assay.

**FIG 8:**
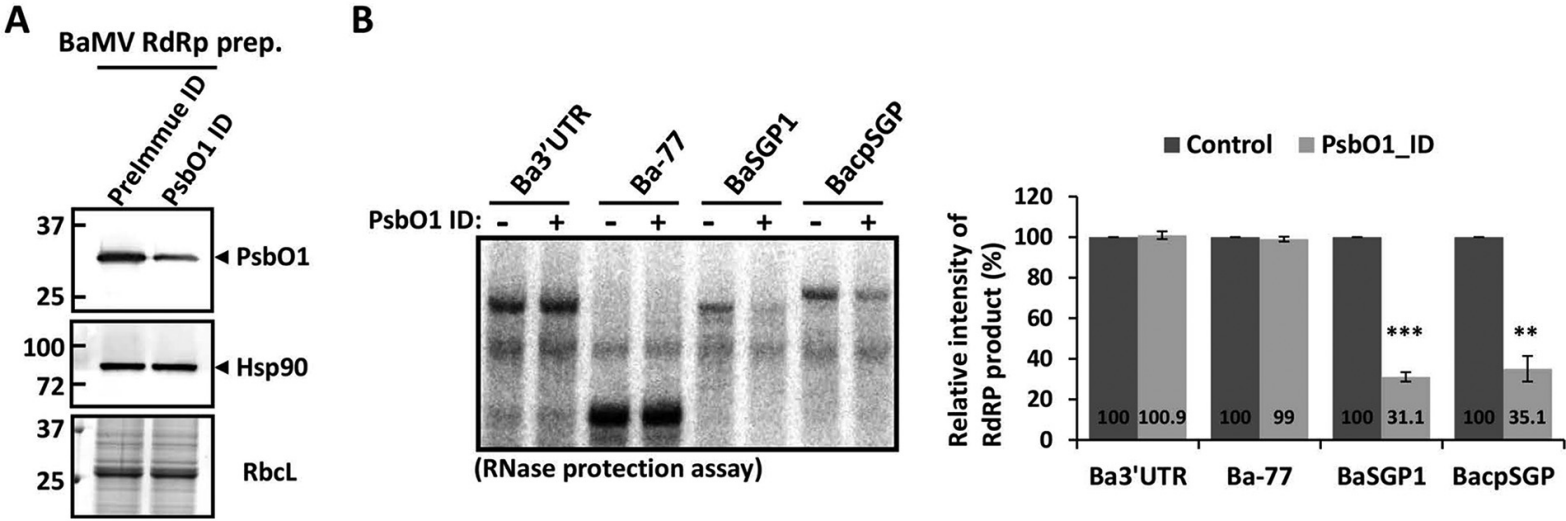
*In vitro* RNA synthesis assay with the NbPsbO1-depleted RdRp preparation. (A) Immunoblot analysis of PsbO1 and Hsp90 in the NbPsbO1-depleted BaMV RdRp preparation. Immunodepletion (ID) was performed with preimmune or PsbO1 antibody. Total proteins from the BaMV RdRp preparation after preimmune ID (control) or PsbO1 ID were separated on a 12% SDS-PAGE gel and then stained with Coomassie blue or probed with antiserum against PsbO1 and Hsp90. (B) Analysis of BaMV RNA synthesized *in vitro* by BaMV RdRp preparation after preimmune ID (−) or PsbO1 ID (+). RdRp assays were done with various transcripts, as indicated above each lane, added exogenously as templates. An RNase protection assay was performed to demonstrate the double-stranded RNA (dsRNA) product. ^32^P-labeled products were analyzed by electrophoresis with a 5% polyacrylamide gel, followed by autoradiography. The right panel shows the quantification of the relative signals of RdRp products, derived from the left panel. Data are the mean ± SD of the results from at least three experiments (Student's *t* test: **, *P* < 0.01; ***, *P* < 0.001).

The above results showed that NbPsbO1 can facilitate the accumulation of BaMV sgRNAs in N. benthamiana. Further validation of this observation requires an *in vitro* RdRp assay with exogenous RNA templates and different concentrations of NbPsbO1. To reduce the protein concentration of endogenous NbpsbO1, the purified BaMV RdRp preparation was subjected to immunodepletion (ID) with preimmune or anti-PsbO1 antibody. Removal of NbPsbO1 was quantified by Western blot analysis to reach about 70% to 80% (Fig. 8A). However, PsbO1 ID could not alter Hsp90 accumulation in BaMV RdRp preparations, so PsbO1 and Hsp90 might exist in the RdRp complexes without direct interaction (Fig. 8A). *In vitro* RdRp assays were then performed with a control or the PsbO1 ID preparation along with the addition of various RNA templates, representing different promoter regions. Depletion of PsbO1 could reduce the RNA synthesis of BaSGP1 and BacpSGP to 31% and 35%, respectively, with no significant effects observed for the synthesis of Ba 3′UTR and Ba-77 (Fig. 8B). These data revealed that NbPsbO1 is specifically required for efficient BaMV sgRNA synthesis but not for (−) or (+) BaMV gRNA synthesis.

## DISCUSSION

Viral RNA must participate in at least two major competing processes in the replication cycle. (i) Viral translation and replication use the same viral (+)RNA template, proceeding in opposite directions. (ii) Synthesis of progeny RNA and sgRNA use the same viral (−)RNA template with distinct promoters. These processes should be highly regulated and compartmentalized to avoid conflict (1). The involvement of host factors in the regulation of switch between translation and replication with viral (+)RNA templates has been reported in previous studies. For example, the decapping activator Lsm1P-7P/Pat1p/Dhh1p complex involved in mRNA degradation has been shown to be a key regulator in the switch from translation to replication of *Brome mosaic virus* (BMV) RNAs by refolding BMV RNAs, resulting in the separation of ribosomes and translation factors and the association with BMV replicase and host proteins for replication (41, 42). A component of *Hepatitis C virus* (HCV) RNA replication complex, polypyrimidine tract-binding protein (PTB), has also been shown to bind to the internal ribosome entry site (IRES) of HCV RNA and inhibit its translation. PTB has also been shown to play a central role in HCV RNA replication by binding to the poly(U/C) tract at the 3′ untranslated region of HCV RNA, which suggests that PTB is involved in the switch from translation to replication of HCV RNA (43, 44). In contrast, to date, the information on the host proteins involved in the regulation between the synthesis of gRNA and sgRNA is still limited. The protein abundance of heterogeneous nuclear ribonuclear protein K (hnRNP K) was significantly increased after *Sindbis virus* (SIN) infection. HnRNP K coprecipitated with the sgRNA but not the gRNA of SIN. The interaction of hnRNP K with SIN sgRNA might upregulate the sgRNA synthesis that produces more sgRNA than gRNA (45). However, the role of hnRNP K during SIN infection is still undetermined. The PTB was identified via the interaction with transcription-regulating sequence of *Mouse hepatitis virus* (MHV) (46). However, the role of PTB in MHV replication and transcription is not clear (47). In this study, we used the RNA fragment of BaMV sgRNA promoter as bait and identified NbPsbO1 as a novel host factor associated with BaMV transcription complexes. NbPsbO1 can interact with the BaMV sgRNA promoter specifically (Fig. 2) and is required for efficient accumulation of BaMV RNA, as demonstrated by the use of TRV-based VIGS (Fig. 3) and the immune-depletion RdRp assay (Fig. 8). In contrast, NbPsbO1 did not bind to promoter sequences for the synthesis of (−)RNA and (+)RNA of BaMV, nor was it required for the synthesis of corresponding products in a cell-free system with antibody-based depletion of NbpsbO1 (Fig. 8). These findings reveal a new role of NbPsbO1 as a specific interaction partner of BaMV RNA involved in the differential regulation of gRNA and sgRNA synthesis. Also, according to the binding patterns from the UV cross-linking assay, BaMV replication and transcription may utilize different sets of host factors for regulating different RNA syntheses (Fig. 2A).

**FIG 9:**
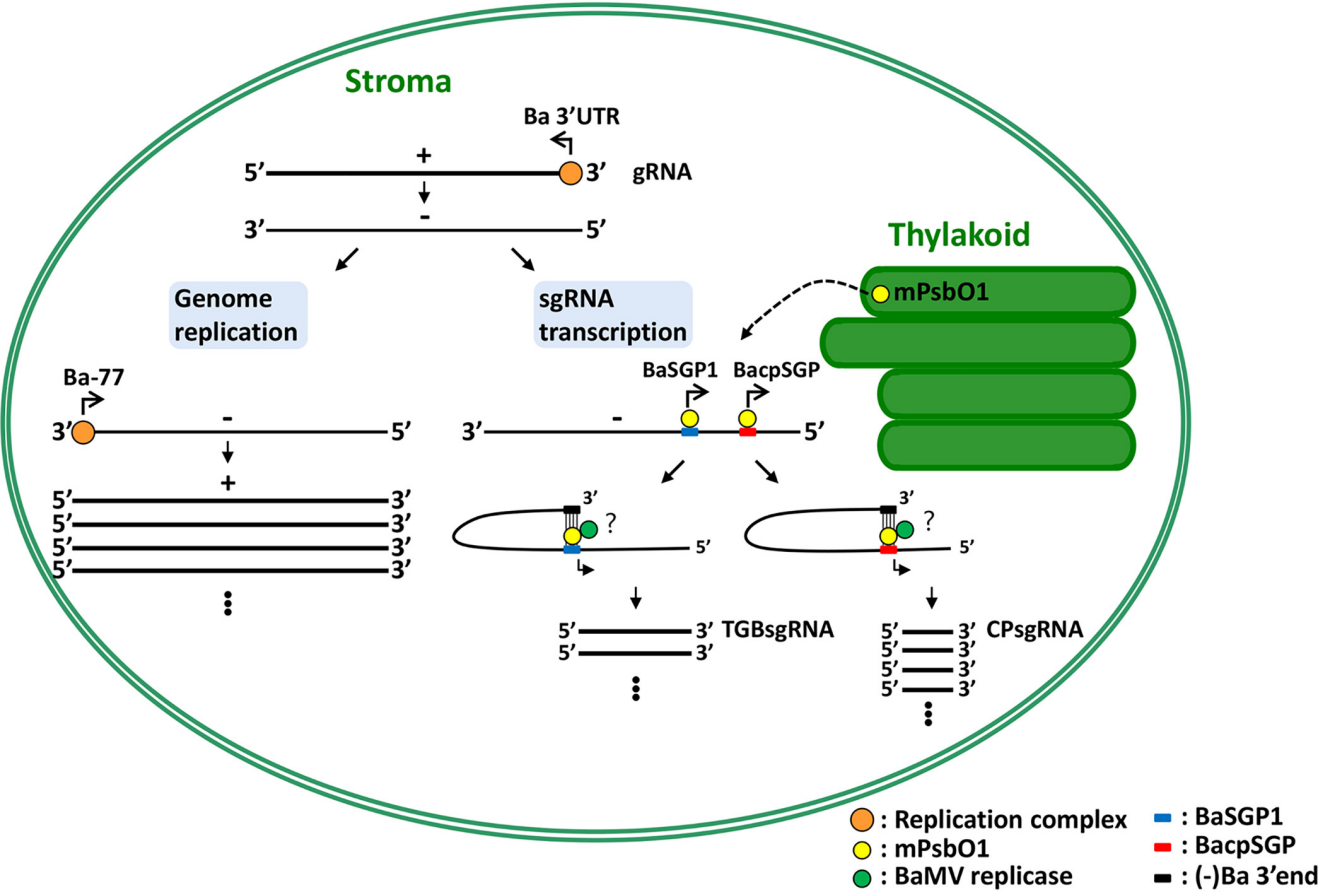
Schematic representation of the model for BaMV RNA-replicase/host factor interaction in the replication and transcription steps. BaMV replication and transcription take place in chloroplast stroma. BaMV replication initiates from the recognition of Ba 3′UTR by replication complexes, followed by BaMV negative-stranded (−)RNA synthesis. The BaMV (−)RNA is a template for gRNA replication and for sgRNA transcription. In replication, a similar set of replication complexes recognize Ba-77 for synthesis of BaMV progeny RNA asymmetrically. In transcription, the mature NbPsbO1 (mPsbO1) is retargeted to stroma and binds to promoter regions for the synthesis of TGBsgRNA and CPsgRNA, namely, BaSGP1 and BacpSGP, respectively. The long-distance RNA-RNA interactions that occur between the (−) BaMV 3′ end and BaSGP1 or between the (−) BaMV 3′ end and BacpSGP control TGBsgRNA and CPsgRNA synthesis, respectively. However, the recognition of BaMV replicase to the subgenomic promoter-like sequences (SGPs) via PsbO1 is not known.

Viral transcription relies on *cis*-acting RNA elements containing a functional sequence and structure to direct the virus-encoded RdRp and/or cellular host proteins to assemble transcription complexes (6–8, 48, 49). The *cis*-acting elements required for sgRNA synthesis were examined by using the satBaMV expression cassette (25) and this study. The minimum sequence required for CPsgRNA synthesis folded into SLs in the negative strand. Maintaining the integrity of the SL2 structure and the conserved octamer motif (3′-CAAUUCAA-5′) in the loop were essential for CPsgRNA synthesis (Fig. 2H) (25). The negative strand of the *cis*-acting element for TGBsgRNA synthesis was predicted to have SLs similar to those of BacpSGP. The conserved octamer motif (3′-CAAUUCAU-5′) is also located in the loop of SL2 (Fig. 2G). NbPsbO1 might interact with such a particular sequence and/or structure to assist in the initiation of BaMV transcription, and the binding affinities of NbPsbO1 to BaMV SGPs are also related to the transcription activities (Fig. 2I and J). The long-distance RNA-RNA interaction between the conserved octamer motif and the 3′-terminal sequence of (−) gRNA was required for transcription of *Potato virus X* (PVX) sgRNAs (50). We showed complementarity between the octamer motif from the BaMV SGPs and the 3′-terminal sequence of (−) gRNA (Fig. 9), which implies that as with PVX, the long-distance RNA-RNA interaction might also favor the internal initiation mode of BaMV sgRNA synthesis (51). Accordingly, transient overexpression of NbPsbO1 would stabilize such an RNA-RNA interaction that leads to excess BaMV sgRNA transcription, which not only sequestered replicase protein but blocked the (−) 3′-terminal sequence for initiation of (+)RNA synthesis and subsequently inhibited the BaMV gRNA accumulation (Fig. 4C and 7F). Alternatively, because most of the viral replicase does not appear to bind directly to the promoter sequences, the ability to initiate RNA synthesis precisely may depend on interactions with the host proteins that bind directly to the promoter region (9). The results presented here are consistent with this concept that depletion of NbPsbO1 inhibited BaMV replicase for the initiation of sgRNA synthesis but had no effect on the initiation of gRNA synthesis in the cell-free system (Fig. 8B), which indicates that NbPsbO1 might recruit BaMV replicase and cooperate to form transcription complexes on BaMV SGPs. However, the interaction between NbPsbO1 and BaMV replicase needs to be further verified (Fig. 9). Moreover, the depletion of NbpsbO1 did not alter the protein abundance of Hsp90, which participates in BaMV replication complexes (29), in the BaMV RdRp preparation (Fig. 8A). Thus, again, BaMV may assemble replication complexes and transcription complexes with different host factors.

BaMV replication in chloroplasts was supported by several lines of evidence, including the following. (i) The nucleus-encoded chlPGK interacts with BaMV 3′UTR and moves the viral RNA into chloroplasts for replication (10, 52). (ii) BaMV RNA was detected and visualized in chloroplasts. *In situ* hybridization and immunogold-labeling experiments revealed BaMV genomic RNA in chloroplasts of BaMV-infected leaf tissues of green bamboo (53). In addition, an MS2 RNA tagging assay revealed BaMV genomic RNA in chloroplasts of N. benthamiana protoplast cells (52). Moreover, BaMV (−)RNA, representing the replication intermediate, was detected in chloroplasts isolated from BaMV-infected N. benthamiana by reverse transcription PCR (RT-PCR) (52). 3) The chloroplast Hsp70 was coprecipitated with BaMV replicase and involved in associating BaMV replication complexes with chloroplast stroma for replication (26). In the present study, we provide further evidence that a nucleus-encoded and chloroplast-targeted protein, NbPsbO1, was identified in association with the BaMV transcription complex and shown to interact with BaMV SGPs (Fig. 2). Confocal microscopy revealed that NbPsbO1 translocated into the thylakoid lumen in chloroplasts by a bipartite transit peptide (Fig. 5B and C) (54). Of note, NbPsbO1 in the BaMV-infected cells was relocalized into the chloroplast stroma (Fig. 5D to F). The protein molecular weight detected in the stroma fraction was the same as that of the mature form detected in the thylakoid fraction, implying that BaMV infection may lead to the release of the NbPsbO1 from the thylakoid lumen to the stroma, instead of that BaMV infection blocks the entry of NbPsbO1 to the thylakoid lumen and sequesters it in the stroma. These results strongly suggest that NbPsbO1 is recruited into the BaMV transcription complex via the interaction with BaMV SGPs. Although the mechanism underlying this relocalization of NbPsbO1 remains unsolved, we speculate that the timing of NbPsbO1 retargeting might also play a role in the regulation of BaMV replication and transcription.

PsbO, PsbP, and PsbQ are extrinsic OEC protein subunits and are involved in the photooxidation of water during PSII (31). OEC has a key role in stabilizing the active manganese site and locates at the thylakoid membrane-lumen interface (55–57). OEC proteins have been reported to interact with various viral proteins and are involved in distinct steps in the life cycle of virus infection. For instance, PsbP protein was shown to interact with CP of *Alfafa mosaic virus* (AMV) or disease-specific protein of *Rice stripe virus* (RSV). Overexpression of PsbP led to repression of AMV and RSV accumulation (39, 58). A geminivirus beta-satellite-encoded βC1 protein interacts with PsbP and reduces PsbP‐mediated antiviral defense (59). NbPsbO interacts with replicase of *Tobacco mosaic virus* (TMV) and inhibits TMV replication in plants (60). NbPsbO1 also interacts with potyvirus 6K2 protein and positively regulates *Tobacco vein banding mosaic virus* (TVBMV) replication (61). The proper function of OEC in PSII produces defense-related reactive oxygen species (ROS) against a pathogen (62). Conversely, downregulation of *NbPsbO* expression suppressed the replication of TVBMV and *Potato virus Y* (61). The potyvirus 6K2 protein hijacks NbPsbO to the induced chloroplast-bound VRC for potyvirus replication (61). To date, however, there has been no previous evidence of a direct interaction between NbPsbO and viral RNA or of the involvement of such an interaction in regulating viral RNA synthesis. In this study, we provide evidence demonstrating the novel function and requirement of NbPsbO1 in the specific regulation of BaMV sgRNA synthesis through direct interaction with RNA templates (Fig. 2).

In summary, with insights from current and previous studies, we propose a hypothetical model to illustrate the stages at which NbPsbO1 participates in BaMV RNA transcription. This model also highlights the differential requirement of NbPsbO1 for synthesis of BaMV gRNA and sgRNAs (Fig. 9). Once BaMV enters a host cell, the RNA genome is used as a template for translation of the replicase. The nucleus-encoded chlPGK interacting with Ba 3′UTR plays a role in ushering the viral RNA and its associated proteins, including replicase, into chloroplast stroma for replication (10, 52). Additionally, Hsp90 and glutathione transferases (NbGSTU4) interact with BaMV 3′UTR and assist in proper assembly of BaMV replication complexes in an ROS elimination environment (29, 63). BaMV replication initiates from the recognition of Ba 3′UTR by replication complexes followed by BaMV (−)RNA synthesis. The BaMV (−)RNAs are templates for both genome replication and sgRNA transcription. For replication, a similar set of replication complexes directly recognize the 3′ end of newly synthesized BaMV (−)RNA (Ba-77) for synthesis of BaMV (+)gRNA asymmetrically (23). For transcription of sgRNAs, BaMV SGPs on BaMV (−)RNA, containing particular sequences and structures (Fig. 2G and H), recruit NbPsbO1 from the thylakoid via physical interactions, which might be required for efficient transcription of BaMV sgRNAs. The long-distance RNA-RNA interactions between the 3′-terminal sequence of BaMV (−)gRNA and distinct octamer motifs might sequester Ba-77 from recognition by replication complexes, thus switching to transcript-corresponding BaMV sgRNAs internally. Although the recognition of replicase to the transcription complexes by NbPsbO1 needs to be further elucidated, depletion of NbPsbO1 specifically interferes with sgRNA synthesis *in vitro* (Fig. 8), which strongly suggests that NbPsbO1 is required for transcription of BaMV sgRNAs. Our study provides insight into the host factor directing sgRNA promoter recognition, which will hasten our efforts to uncover the mysteries of viral RNA replication and transcription.

## MATERIALS AND METHODS

### Construction of BaMV infectious clones.

To construct pKBRepHA_MluI_, we used a two-step procedure. In the first step of the construction of pCBRepHA_MluI_, a megaprimer was synthesized by a first PCR amplification with pCB-RepHA (26) as the template, with the primer pairs for Ba4203RCGC (5′-CTAATAGGTTACGCGTTATGCGTAATC-3′) and Ba3572F (5′-ATGAAAGCAAGGCACCATG-3′). The purified megaprimer was used in the second PCR amplification, with pCB-RepHA as a template and a reverse primer, Ba5361R (5′-GGGCAGATGCTGTTGAAG-3′). The products of the second PCR were gel purified, digested with AflII and NsiI, and used to replace the corresponding fragment within the pCB-RepHA plasmid after digestion with the same restriction enzymes to generate pCBRepHA_MluI_. To construct pCBRepHA21, pCBRepHA50, and pCBRepHA80, the PCR products were amplified with PCB, an infectious clone of BaMV-S, as the template, with the forward primers of MluI/Ba4171 (5′-GCACGCGTGAAGGTTTATTCTC-3′), MluI/Ba4142 (5′-GCACGCGTCGCCAACCACGATG-3′), and MluI/Ba4112 (5′-GCACGCGTCCCTACATACAGAAG-3′), respectively, and reverse primer Ba5361R. The purified PCR products were digested with MluI and NsiI and used to replace the corresponding fragment within pCBRepHA_MluI_ after digestion with the same restriction enzymes to generate pCBRepHA21, pCBRepHA50, and pCBRepHA80, respectively. The second step was to introduce these plasmids into the Agrobacterium tumefaciens binary vector pKn (37). Plasmids pCBRepHA_MluI,_ pCBRepHA21, pCBRepHA50, and pCBRepHA80 were digested with SbfI and SacI and then ligated into the pKn vector to generate plasmids pKBRepHA_MluI,_ pKBRepHA21, pKBRepHA50, and pKBRepHA80, respectively.

### Plant inoculation by agroinfiltration.

Plasmids pKB (64), pKBRepHA_MluI,_ pKBRepHA21, pKBRepHA50, and pKBRepHA80 were introduced into A. tumefaciens strain GV3850 individually by electroporation. A. tumefaciens cultures were collected by centrifugation and resuspended in infiltration buffer (10 mM MES [morpholineethanesulfonic acid] buffer, pH 5.5, and 10 mM MgCl_2_); suspensions were adjusted to an optical density at 600 nm (OD_600_) of 0.1 and infiltrated by syringe into the leaves of each test plant.

### BaMV RNA analysis by Northern blot hybridization.

Total RNA was extracted from inoculated leaves by using TriPure isolation reagent (Roche Life Science, Germany). RNA samples were separated by electrophoresis and transferred to nylon membranes (Amersham, Little Chalfont, UK) for Northern blot analysis (21). Blots were hybridized with a riboprobe specific for BaMV 3′UTR. The ^32^P-labeled probe was transcribed from HindIII-linearized pBaHB by using SP6 RNA polymerase (53).

### Protein analysis and antibodies.

Total protein was extracted from leaf tissue, and protein samples were separated by 12% or 10% SDS-PAGE. For Western blot analysis, separated proteins were transferred to a polyvinylidene difluoride (PVDF) membrane (Millipore, USA) and incubated with laboratory-generated primary antiserum from rabbits against BaMV TGBp1 (65), NbPsbO1, NbHsp90, or actin at a 1:5,000 dilution.

### *In vitro* RdRp assay.

The replication complexes of BaMV used for an *in vitro* RdRp assay were purified from BaMV-infected N. benthamiana plants as described previously (66). To analyze the exogenous template activity, the membrane-bound BaMV RdRp complexes were solubilized with 1.5% NP40 and treated with micrococcal nuclease to digest endogenous templates. The RNA templates of Ba 3′UTR, Ba-77, and CMV 3′UTR with lengths of 186, 77, and 263 nt, respectively, for RdRp assays were described previously (23, 28). Short fragments containing a T7 promoter and sequences derived from the full-length infectious cDNA clone pCB were generated by PCR amplification for minus-strand RNA synthesis. Minus-strand RNA transcripts with promoter regions for synthesis of TGBsgRNA (BaSGP1) or CPsgRNA (BacpSGP) were synthesized from the PCR-amplified DNA fragments with primer pair Ba4142 (5′-TCGCCAACCACGATGCAATC-3′) and T7Ba(−)4312 (5′-TAATACGACTCACTATAGGGCACCAGCCACCGCGTGC-3′) for BaSGP1 and primer pair Ba5391 (5′-GAAATAATAATAAACGGGC-3′) and T7Ba(−)5617 (5′-TAATACGACTCACTATAGGGCTGGGCTGCAGCTTG-3′) for BacpSGP. The method for exogenous RdRp activity assay was as previously described (28), except that 200 ng of transcript RNAs was added to the reaction mixture. The RNase protection assay was performed as described previously (28).

### UV cross-linking assay.

The cross-linking assay was performed as described previously (29), except that fractions 5 to 7 of RdRp preparations of the sucrose gradient were pooled and treated with micrococcal nuclease and incubated with 20 fmol of [α-^32^P]UTP-labeled RNA probe along with binding buffer (20 mM Tris-HCl [pH 8.0], 3 mM MgCl_2_, 10 mM KCl, 2 mM dithiothreitol [DTT], 4% glycerol) and irradiated with a UV lamp (Stratagene; UV Stratalinker TM 1800) at a 254-nm wavelength on ice for 20 min, followed by RNase A (10 μg) and RNase T1 (0.5 U) digestion at 37°C for 30 min. The UV cross-linked products were separated on a 12% SDS-PAGE gel. The gel was dried and observed under a phosphorimager. The protein bands corresponding to RNA-protein complexes were excised from the gel and subjected to liquid chromatography-tandem mass spectrometry (LC/MS-MS).

### Expression and purification of recombinant protein Trx-His-mPsbO1.

A cDNA fragment corresponding to the mature form of the NbPsbO1 open reading frame was amplified from the primers KpnI/NbPsbO1_256F (5′-GCGGTACCGAAGGAGCTCCAAAACGTCTAAC-3′) and BamHI/NbPsbO1_cdsR (5′-GCGGATCCTCATTCAAGTTGGGCATACC-3′) by RT-PCR with N. benthamiana total RNA as a template. The gel-purified PCR products were digested with KpnI and BamHI and ligated to pET32a to create the expression vector pET32a-mPsbO1. The vectors pET32a and pET32a-mPsbO1 were introduced into Escherichia coli BL21 cells, and the control protein (Trx-His) and recombinant fusion protein (Trx-His-mPsbO1) were induced and purified per the manufacturer’s recommendations (YEA-His minikit; Yeastern Biotech).

### Electrophoretic mobility shift assay (EMSA).

RNA probes were synthesized by using T7 RNA polymerase-based transcription in the presence of ^32^P-labeled UTP. Various amounts of affinity-purified recombinant protein Trx-His-mPsbO1 was incubated with radioactively labeled RNA probe (20 fmol) in a binding buffer (20 mM Tris-Hcl [pH 8.0], 3 mM MgCl_2_, 10 mM KCl, 2 mM DTT, 4% glycerol) at room temperature for 15 min. The reaction mixtures were subjected to electrophoresis on a 5% nondenaturing polyacrylamide gel, and the RNA-protein complexes were detected by a phosphorimager (GE Amersham Typhoon). For the competition assays, various amounts of unlabeled competitor RNA were preincubated with 2 μg of Trx-His-mPsbO1 for 15 min prior to the addition of ^32^P-labeled BacpSGP probe. For the preparation of BacpSGP mutants as competitors, the plasmids pyTABacpSGPM1 and pyTABacpSGPM2 harboring the regions of the BacpSGP mutants were constructed by two-step PCR amplification from pCB. The first PCR amplification was done with primer pair Ba5391 and cpSGPM1_R (5′-CAAAAGAAGGAATAAATCCAAACCCTAG-3′) for BacpSGPM1 and primer pair Ba5391 and cpSGPM2_R (5′-GTTTAATTTACAAAAGTACCAAACTTAAC-3′) for BacpSGPM2 purified as megaprimers. The megaprimers were used in the second PCR amplification with pCB as a template and a reverse primer, T7Ba(−)5617. The PCR products were gel purified and individually cloned into vector pyT&A. RNA competitors were prepared by *in vitro* transcription with the EcoRI-linearized plasmids with T7 RNA polymerase.

### VIGS.

*Tobacco rattle virus* (TRV)-based virus-induced gene silencing (VIGS) was used to knock down the expression of *NbPsbO1* (35). Plasmids pTRV1 and pTRV2-Luc were kindly provided by David C. Baulcombe (Department of Plant Sciences, University of Cambridge, UK). For the construction of pTRV2-NbPsbO1, a 500-bp fragment of the NbPsbO1 coding sequence from N. benthamiana was amplified by PCR with N. benthamiana cDNA and gene-specific primers XbaI/NbPsbO1_VIGSF (5′-GCTCTAGACCCCAGATTTCCAGAAAACTAAG-3′) and BamHI/NbPsbO1_VIGSR (5′-CGGGATCCCACATCCTTGGGGACCTTTG-3′). The amplified PCR products were gel purified, digested with XbaI and BamHI, and cloned into the pTRV2 plasmid (35) after digestion with the corresponding restriction enzymes to generate pTRV2-NbPsbO1. pTRV1, pTRV2-Luc, and pTRV2-NbPsbO1 were individually transformed into A. tumefaciens strain C58C1 for knockdown experiments. A. tumefaciens cultures (OD_600_, 0.2) containing pTRV2-Luc or pTRV2-NbPsbO1 was mixed with pTRV1-containing C58C1 at a 1:1 volume ratio and coinfiltrated by syringe onto three leaves of each test plant. At 10 days postagroinfiltration (dpai), the third and fourth leaves above the infiltrated leaves were isolated by using TriPure isolation reagent (Roche Life Science, Germany), per the manufacturer’s instructions. For measuring the knockdown efficiency of *NbPsbO1* in plants, real-time quantitative RT-PCR was used with the primers, PsbO1-qF (5′-GACCGGTGAGGTCATTGGAG-3′) and PsbO1-qR (5′-CAATGCTTGTCCAAATTGAG-3′). To normalize the mRNA levels of target genes between samples, the relative mRNA levels of actin were determined with the primers actin-F (5′-GATGAAGATACTCACAGAAAGA-3′) and actin-R (5′-GTGGTTTCATGAATGCCAGCA-3′). At 10 dpai, A. tumefaciens cultures containing pKBG (37) (OD_600_, 0.1) were infiltrated into the third and fourth leaves above the TRV-infiltrated leaves. Total RNA was extracted from the BaMV-inoculated leaves of individual plants 2 dpai.

### Protoplast isolation and virus infection.

Protoplasts were isolated from N. benthamiana leaves as previously described (22). For each inoculation, 0.5 μg of viral RNA or 10 μg of plasmid DNA of infectious cDNA clone was used to inoculate 2 × 10^5^ protoplasts. To construct pCBSGPM, a megaprimer was synthesized by a first PCR amplification with pCB as the template, with the primer pair Ba5053 (5′-TACCCTTCCACACACCGGCG −3′) and cpSGPM_R (5′-GTTTAATTTACAAAAGTACCTTTCGTAAGAAACCCTAGCTGGAG-3′). The purified megaprimer was used in the second PCR amplification with pCB as a template and a reverse primer, pCass3′ (5′-AGAGAGACTGGTGATTTCAG-3′). The products of the second PCR were gel purified, digested with NsiI and SacI, and used to replace the corresponding fragment within the pCB-RepHA (26) after digestion with cognate restriction enzymes to generate pCBSGPM.

### Transient expression of NbPsbO1 and NbPsbO1 mutants.

To overexpress PsbO1 and PsbO1 mutants in N. benthamiana, the plasmids pBIPsbO1-T7, pBIdTPPsbO1-T7, and pBIsPsbO1-T7 were generated. The coding sequences (CDSs) for PsbO1-T7 and dTPPsbO1-T7 were amplified by PCR with N. benthamiana cDNA and primer pairs (for PsbO1-T7, XbaI/NbPsbO1_cdsF [5′-GCTCTAGAATGGCTGTCTCTTTACAAG −3′] and XhoI/NbPsbO1 T7_cdsR [5′-GCCTCGAGTCAACCCATTTGCTGTCCACCAGTCATGCTAGCCATTTCAAGTTGGGCATACC-3′]; for dTPPsbO1-T7, XbaI/dTPNbPsbO1_cdsF [5′-GCTCTAGAATGGAAGGAGCTCCAAAACGTCTAAC-3′] and XhoI/NbPsbO1 T7_cdsR). PCR products were gel purified, digested with XbaI and XhoI, and used to replace the corresponding fragment within pBI-mGFP (52) after digestion with the same restriction enzymes to generate pBIPsbO1-T7 and pBIdTPPsbO1-T7, respectively. For constructing pBIsPsbO1-T7, a megaprimer was synthesized by a first PCR from the pBIPsbO1-T7 template with primer pairs XbaI/NbPsbO1_cdsF and PsbO1_CTP_only (5′-CGTTTTGGAGCTCCTTCCAAGTCCTTGAG-3′). The purified megaprimer was used in the second PCR with pBIPsbO1-T7 as a template, with the primer for XhoI/NbPsbO1 T7_cdsR. PCR products were gel purified, digested with XbaI and XhoI, and used to replace the corresponding fragment within pBI-mGFP to generate pBIsPsbO1-T7. The pBI-based plasmids for transient expression of NbPsbO1-T7, NbdTPPsbO1-T7, and NbsPsbO1-T7 were introduced into A. tumefaciens strain GV3850 individually by electroporation. A. tumefaciens cultures were collected by centrifugation and resuspended in infiltration buffer. Suspensions were adjusted to an OD_600_ of 0.5 and infiltrated by needleless syringe into the leaves of each test plant.

### Subcellular localization by confocal microscopy.

To visualize the localization of NbPsbO1 and NbPsbO1 mutants in N. benthamiana protoplast cells, plasmids pEPsbO1-OFP, pEdTPPsbO1-OFP, and pEsPsbO1-OFP were generated. The CDSs for PsbO1 and dTPPsbO1 were amplified by PCR from the pBIPsbO1-T7 template with primer pairs (for PsbO1, XbaI/NbPsbO1_cdsF and BamHI/NbPsbO1_cdsR2 [5′-GCGGATCCTTCAAGTTGGGCATACC-3′]; for dTPPsbO1, XbaI/dTPNbPsbO1_cdsF and BamHI/NbPsbO1_cdsR2). PCR products were gel purified, digested with XbaI and BamI, and cloned into pEpyon-OFP (26) to generate pEPsbO1-OFP and pEdTPPsbO1-OFP, respectively. For constructing pEsPsbO1-OFP, the CDSs for sPsbO1 were amplified by PCR from the pBIsPsbO1-T7 template with primer pairs XbaI/NbPsbO1_cdsF and BamHI/NbPsbO1_cdsR2. PCR products were gel purified, digested with XbaI and BamI, and cloned into pEpyon-OFP to generate pEPsbO1-OFP and pEdTPPsbO1-OFP, respectively. The amplified DNA fragment was gel purified, digested with XbaI and BamI, and cloned into pEpyon-OFP. The A. tumefaciens GV3850 strain harboring pEpyon-OFP, pEPsbO1-OFP or its derivatives, pEPsbP4-GFP, or pt-gk (40) was infiltrated into N. benthamiana leaves. Three days later, images of protoplasts were obtained under an inverted fluorescence confocal microscope (FV1000; Olympus) with 543-, 633-, and 488-nm laser excitations for OFP, chloroplast autofluorescence, and GFP imaging, respectively.

### Chloroplast isolation and fractionation.

The chloroplast isolation was done as described previously (52). The intact chloroplasts were isolated from the interface layer of a Percoll step gradient comprising 40% and 80% Percoll. The isolated chloroplasts were washed three times with 5 ml suspension buffer (0.35 M sorbitol, 10 mM K_2_HPO_4_, 0.5 mM MgCl_2_, 35 mM HEPES-KOH, pH 8.3, and 1 mM dithiothreitol) and saved for total chloroplasts or further suspended in osmotic lysis buffer (20 mM HEPES-KOH, pH 7.5, 10 mM NaHCO_3_, 2 mM MgCl_2_, 2.5 mM EDTA, 2.5 mM EGTA, protease inhibitor) and on ice for 15 min. Supernatant (stroma) and pellet (thylakoid) fractions were separated by centrifugation at 10,000 × *g* for 10 min. The pellet fraction was washed three times in lysis buffer. Stroma proteins were precipitated with ammonium acetate.

### Immunodepletion of NbPsbO1 from BaMV RdRp preparation.

The micrococcal nuclease-treated solubilized BaMV RdRp was centrifuged at 100,000 × *g* for 35 min at 4°C, and the supernatant (S100) was collected for the immunodepletion assay. S100 (0.5 ml) was mixed with 5 μg purified IgG from preimmune or anti-PsbO1 antiserum on a rotamixer for 4 h at 4°C. NbPsbO1 was depleted from S100 by precipitation by adding protein A magnetic beads (GE Healthcare). The samples from preimmune or NbPsbO1-depleted S100 underwent Western blot analyses with antibodies specific to NbPsbO1 or Hsp90.
